# BaalChIP: Bayesian analysis of allele-specific transcription factor binding in cancer genomes

**DOI:** 10.1186/s13059-017-1165-7

**Published:** 2017-02-24

**Authors:** Ines de Santiago, Wei Liu, Ke Yuan, Martin O’Reilly, Chandra Sekhar Reddy Chilamakuri, Bruce A. J. Ponder, Kerstin B. Meyer, Florian Markowetz

**Affiliations:** 1 Cancer Research UK Cambridge Institute, University of Cambridge, Robinson Way, Cambridge, UK; 2 Present Address: Seven Bridges Genomics LTD, UK. 101 Euston Road NW1 2RA, London, UK; 3 Present Address: Institute of Biodiversity Animal Health and Comparative Medicine, University of Glasgow, Glasgow, UK; 4 Present Address: School of Computing Science, University of Glasgow, Glasgow, UK

**Keywords:** Allele-specific binding, ChIP-sequencing, FAIRE-sequencing, Bayesian statistics, Cancer, Copy-number change, Allele frequency

## Abstract

Allele-specific measurements of transcription factor binding from ChIP-seq data are key to dissecting the allelic effects of non-coding variants and their contribution to phenotypic diversity. However, most methods of detecting an allelic imbalance assume diploid genomes. This assumption severely limits their applicability to cancer samples with frequent DNA copy-number changes. Here we present a Bayesian statistical approach called BaalChIP to correct for the effect of background allele frequency on the observed ChIP-seq read counts. BaalChIP allows the joint analysis of multiple ChIP-seq samples across a single variant and outperforms competing approaches in simulations. Using 548 ENCODE ChIP-seq and six targeted FAIRE-seq samples, we show that BaalChIP effectively corrects allele-specific analysis for copy-number variation and increases the power to detect putative cis-acting regulatory variants in cancer genomes.

**Electronic supplementary material:**

The online version of this article (doi:10.1186/s13059-017-1165-7) contains supplementary material, which is available to authorized users.

## Background

Allele-specific measurements of transcription factor (TF) binding from ChIP-seq data have provided important insights into the allelic effects of non-coding variants and their contribution to phenotypic diversity [1–5]. Since the majority of disease-risk-associated single-nucleotide polymorphisms (SNPs) occur in non-coding DNA, many of them might disrupt cis-regulatory elements. Thus, a direct method of identifying functional SNPs is to use the information obtained from allelic-specific binding (ASB) of TFs. ChIP-seq data are commonly used to study ASB of proteins at heterozygous non-coding SNPs and to infer putative effects of such variants on gene regulation. Allele-specific mapping corrects for environmental sources of variation that alter gene regulation, since both alleles are assayed in the same cellular context.

### Existing approaches to infer allelic imbalance

Previous studies have used ChIP-seq and RNA-seq to identify ASB and allele-specific expression (ASE). These studies have described methods to address technical and methodological biases such as the sequence context of a SNP [6], alignment biases to the reference allele [7, 8], and the issue of increasing detection power by combining multiple SNPs in the same gene [9] or across multiple ChIP-seq samples [10]. However, all of these approaches are designed to examine the allelic imbalances in diploid samples and do not address copy-number differences between the two alleles, a ubiquitous feature of cancer genomes (Additional file 1: Figure S1).

Few papers in the literature have tried to address confounding effects arising from copy-number changes. Some studies have analyzed tumor and normal samples without making any adjustments to the applied methodology, which risks identifying false positives where the detected ASE is mainly due to copy-number alterations [11]. Others have removed all sites overlapping copy-number variants [7, 12, 13], or used the genomic DNA (gDNA) allelic ratios (ARs) to correct for the observed allelic imbalances [14, 15] or to remove SNPs with allelic imbalances detected in the control input DNA [2], which is feasible only when the coverage at any assayed heterozygous site is high (>20 × if a binomial test is to be applied to reach adequate statistical power [9, 12]). Recently, Liu et al. [16] developed cisASE, a likelihood-based method for detecting ASE. cisASE has been shown to correct successfully for copy-number alterations that hinder the identification of ASE in RNA-seq data. Additional file 2: Table S1 presents a summary of the different strategies of allelic-specific mapping analyses to deal with regions of altered copy number. While the analysis of ASE and ASB are conceptually similar, in practice, methods tailored to either ASE or ASB are not easily exchangeable, because the data types and, thus, the biological and statistical assumptions are different.

### Bayesian analysis of ASB

**Fig. 1:**
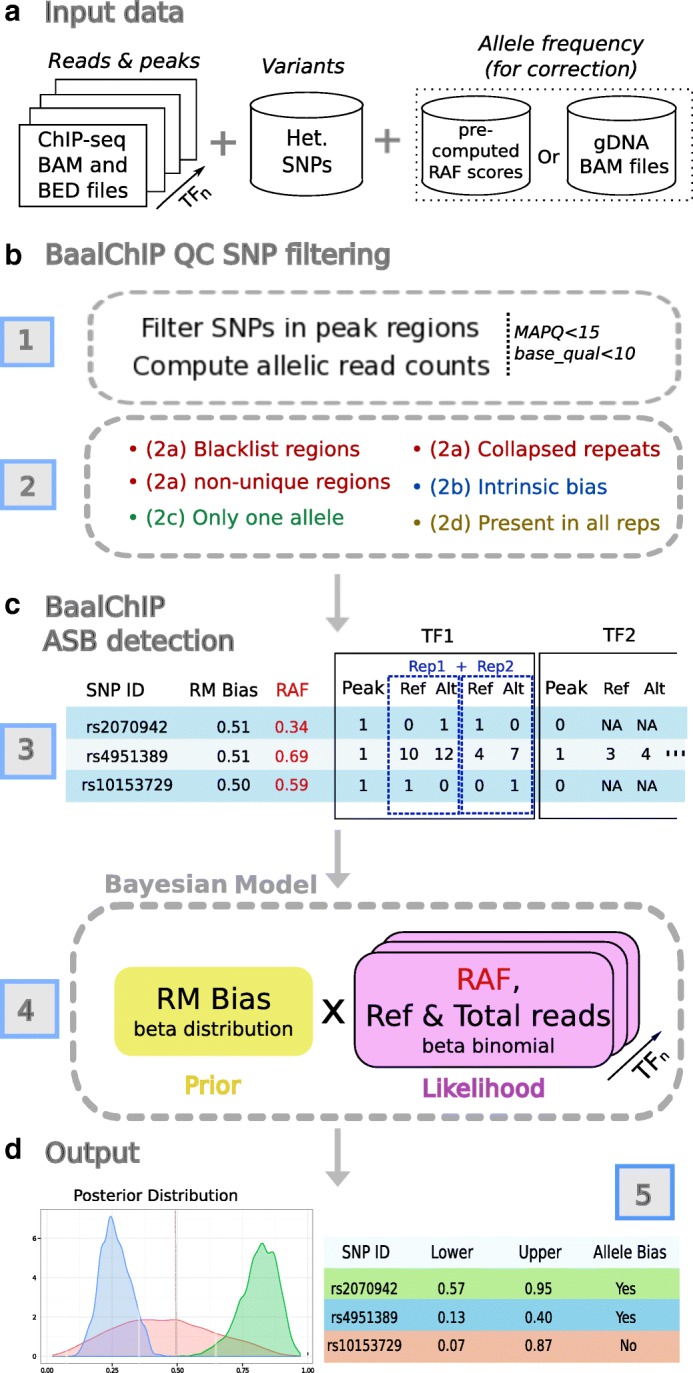
Description of BaalChIP model. **a** The basic inputs for Baal are the ChIP-seq raw read counts in a standard BAM alignment format, a BED file with the genomic regions of interest (such as ChIP-seq peaks), and a set of heterozygous SNPs in a tab-delimited text file. Optionally, genomic DNA BAM files can be specified for RAF computation. Alternatively, the user can specify the pre-computed RAF scores for each variant. **b** The first module of BaalChIP consists of (*1*) computing allelic read counts for each heterozygous SNP in peak regions and (*2*) a round of filters to exclude heterozygous SNPs that are susceptible to generating artifactual ASB effects. (*3*) The reference mapping (RM) bias and the reference-allele frequency (RAF) are computed internally and the output consists of a data matrix where RM and RAF scores are included alongside information about allele counts for each heterozygous SNP. The column *Peak* contains binary data to indicate the called peaks. **c** The second module of BaalChIP consists of calling ASB binding events. (*4*) BaalChIP uses a beta-binomial Bayesian model to consider RM and RAF bias when detecting ASB events. **d** The output from BaalChIP is a posterior distribution for each SNP. A threshold to identify SNPs with allelic bias is specified by the user (default value is a 95% interval). (*5*) The output of BaalChIP is a credible interval (lower and upper) calculated based on the posterior distribution. This interval corresponds to the true AR in read counts (i.e., after correcting for RM and RAF biases). An ASB event is called if the lower and upper limits of the interval are outside the 0.4–0.6 interval. *Alt* alternative, *AR* allelic ratio, *ASB* allelic-specific binding, *gDNA* genomic DNA, *Het.* heterozygous, *MAPQ*, mapping quality, *NA* not applicable, *RAF* reference-allele frequency, *Ref* reference, *Rep* repeat, *RM* reference mapping, *SNP* single-nucleotide polymorphism, *TF* transcription factor

To address the wide range of biases that can affect the detection of ASB from ChIP-seq data we developed BaalChIP (Bayesian analysis of allelic imbalance from ChIP-seq data) (Fig. 1). BaalChIP makes it possible to quantify the significance of the allelic imbalance from ChIP-seq data in cell lines with genomic aberrations, which is a major advance over previous approaches. BaalChIP combines several important features. First, we use several strategies to account rigorously for allelic mapping bias, including filtering of SNPs in problematic alignment regions as well as simulations to identify reads with an increased risk of mapping bias. Second, we implement a beta-binomial model for allelic read counts. This model accounts for the observed variance in the data being larger than expected from a standard binomial model, a phenomenon known as overdispersion [9, 17, 18]. Third, we take advantage of multiple TF ChIP-seq data, which may be available for the same SNP, to improve ASB detection. Finally, we use a Bayesian framework to account for the influence of the background allele composition and the reference mapping bias on the observed ChIP-seq allelic read count.

We applied BaalChIP to 548 ENCODE samples [19] obtained from a panel of 14 cell lines, representing different tissues and karyotypes, including eight cancer cell lines (HeLa-S3, A549, MCF-7, T-47D, K562, HepG2, SK-N-SH, and HL-60) and six non-cancer cell lines (H1-hESC, GM12878, GM12891, GM12892, MCF10A-Er-Src, and IMR90), as well as six FAIRE-seq samples obtained from breast cancer cell lines (MDA-MB-134 and T-47D) (Additional file 3: Table S2 and Additional file 4: Table S3). We demonstrate that copy-number changes can easily give rise to spurious signals of allelic imbalance. We are able to integrate the quantitative information obtained from ChIP-seq data along with information about the background allele composition to detect ASB accurately and correct for artifacts caused by allele-specific losses or gains at the structural genomic level. Because of this integrated modeling, we were able to detect a large number of ASB events from ChIP-seq data, generating a resource for future functional studies.

BaalChIP is implemented as an R package [20] and it is freely available from the Bioconductor repository (https://bioconductor.org/packages/release/bioc/html/BaalChIP.html) [21].

## Results

### Overview of BaalChIP work flow

In this study, we aim to devise a method that allows us to correct for copy-number changes and other biases in the analysis of ASB from ChIP-seq or similar data. The BaalChIP work flow requires three different sets of input data (Fig. 1): the SNP variants file, ChIP-seq data sets (e.g., sets of BAM and BED files of ChIP-seq data obtained for different proteins that may or may not be grouped into replicates), and a corresponding set of gDNA files for each individual sample. The SNP variants file and the BED files are used to select the sites for the analysis, the ChIP-seq BAM files are used to compute the allelic read counts, and the gDNA files allow us to determine reference-allele frequency (RAF) values to correct for effects of the background allele composition. Alternatively, RAF values may be directly pre-computed from B allele frequency (BAF) scores (Fig. 1 a). It has been shown that removing duplicate reads can reduce technical sources of biases at ASB sites [22] and we suggest that BAM files are preprocessed to contain only uniquely aligned reads or flagged duplicated reads.

#### BaalChIP quality control

The BaalChIP work flow starts by first computing the allelic read counts for each assayed heterozygous SNP overlapping genomic intervals of interest (e.g., ChIP-seq peaks). By default, only uniquely mapped reads (mapping quality >15) and sites with base quality >10 are used (Fig. 1 b). In the next step, several quality control (QC) filters are applied to consider technical biases that may contribute to the false identification of regulatory SNPs (Fig. 1 b).

In a first round, BaalChIP excludes SNPs mapping to regions of known problematic read alignment: blacklisted regions [23], non-unique regions with a genomic mappability score of less than 1 (based on the UCSC mappability track, 50 bp segments) [22], and collapsed repeat regions [24]. The excluded regions contain genomic intervals that are frequently enriched for repetitive elements and frequently cause ambiguous read mapping and sequencing artifacts [25].

The second QC filter performs read-alignment simulations to consider an additional type of read mapping bias named the intrinsic bias [6, 17]. This bias occurs due to intrinsic characteristics of the genome that translate into different probabilities of read mapping. Even when reads differ only in one location, reads carrying one of the alleles may have a higher chance of matching multiple locations (i.e., have many repeats in the genome) and may, therefore, be mapped to an incorrect locus. This, in turn, results in the underestimation of read counts and may cause both false-positive and false-negative inferences of ASB.

The third QC filter selects those SNPs that pass all filters in all replicated samples, provided that replicated samples exist. This step will mainly remove SNPs in regions where the ChIP-seq signal is not consistently detected across all replicates (for instance, when coverage is zero in one of the replicates).

The final QC filter consists of removing possible homozygous SNPs by removing any site where only one allele is observed [4, 26].

#### BaalChIP detection of ASB events

The filtered SNPs and their allelic read counts are merged into a table with the total number of read counts in the reference (Ref) and alternative (Alt) alleles. No data is entered (missing data, NA) if a SNP did not pass the previously applied QC step for that sample (Fig. 1 c).

BaalChIP considers two additional biases that may lead to inaccurate estimates of ASB: the reference mapping (RM) and the RAF biases. The RM bias occurs because the reference genome represents only a single reference allele at each SNP position. Reads that carry the non-reference allele have an extra mismatch to the reference sequence. Previous work has shown that this creates a marked bias in favor of the alignment of reads that contain the reference genome and could, therefore, affect the accuracy of ASB estimates [6].

The RAF bias occurs due to alterations in the background abundance of each allele (e.g., in regions of copy-number alterations) and the correction for this bias is one of the key features of BaalChIP. RAF values at each heterozygous variant are used in the model likelihood to correct the observed ChIP-seq read counts relative to the amount of the reference allele. These are given as relative measures from 0 to 1, where values between 0.5 and 1 denote an underlying bias to the reference allele and values between 0 and 0.5 to the alternative allele. RAF scores can be obtained from SNP array BAFs by assigning the correct reference and alternative alleles to allele A and B generic labels (RAF is equal to BAF if the reference allele corresponds to the B allele; RAF is equal to 1 − BAF if the reference allele corresponds to the A allele). Alternatively, if whole-genome DNA sequencing samples are given as an input, BaalChIP calculates the RAF values directly from the gDNA allelic read counts.

Finally, BaalChIP uses a beta-binomial distribution to model read count data, therefore it accounts for extra variability (overdispersion) in allelic counts, which is often observed in sequencing data [9, 17]. The output of BaalChIP is a posterior distribution of the estimated allelic balance ratio in read counts observed after considering all sources of underlying biases (Fig. 1 c).

### Evaluation of BaalChIP performance

In a controlled simulation study, we compare BaalChIP with the two major competing methods to infer allelic imbalance: the binomial test and iASeq [10]. The binomial test is the method most frequently used to analyze allele specificity from ChIP-seq data [1–4, 7]. In a recent study [15], biases caused by differences in copy number were accounted for by weighting the binomial null with the ARs observed from the gDNA samples (the number of reads mapping to each allele in the gDNA). Therefore, to take this strategy into consideration, we set the null hypothesis on the probability of success to the estimated RAF bias, instead of 0.5. iASeq is another available method that has been shown to improve the detection of ASB [10]. The iASeq method uses a Bayesian framework to combine information from different experiments or replicates, and models the read counts with a beta-binomial distribution, instead of a simple binomial distribution, to account for extra-binomial variation caused by technical variability. Although iASeq was not originally designed to overcome copy-number biases, we included it in our simulation study because of its improvement over the traditional binomial test.

**Fig. 2:**
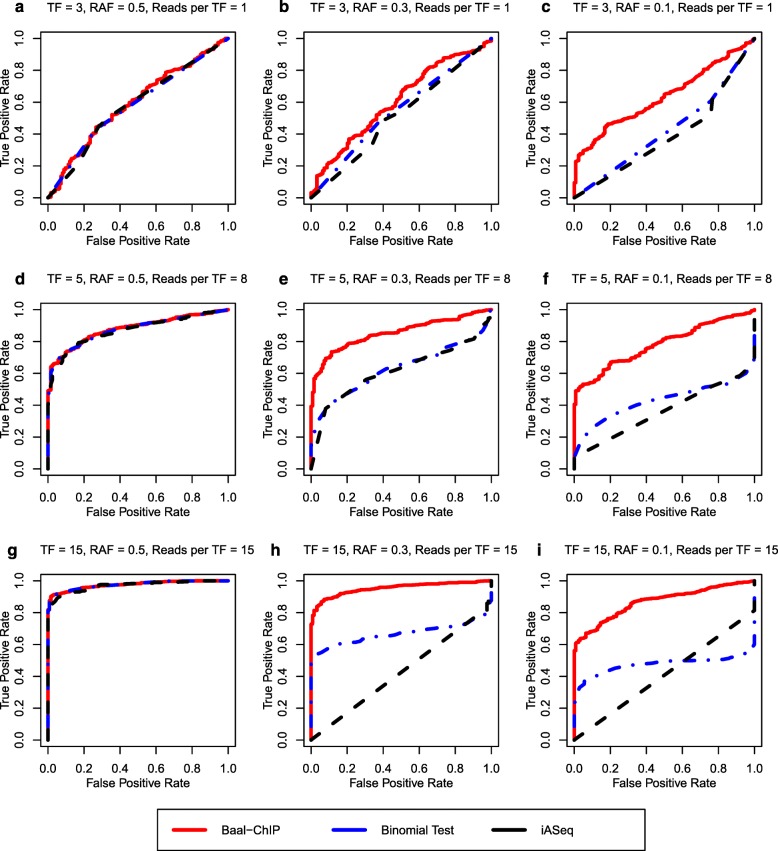
The ROC curve comparison between BaalChIP and other allele-specific SNP finding methods: binomial test and iASeq, using a simulated data set. The BaalChIP result is shown by *solid red line*. Binomial test and iASeq are shown in *dashed blue* and *black lines*. The number of TFs able to bind at a given SNP and the number of reads per TF increases from TF = 3, Reads per TF = 1 (**a**, **b**, **c**) to TF = 5, Reads per TF = 8 (**d**, **e**, **f**) to TF = 15, Reads per TF = 15 (**g**, **h**, **i**). RAF is decreasing from 0.5 (**a**, **d**, **g**) to 0.3 (**b**, **e**, **g**) to 0.1 (**c**, **f**, **i**)

All three methods are tested on synthetic data sets with varying sequencing depth, number of TFs binding to a SNP, and copy-number states. In addition, we also examine the robustness of the methods against a wide range of true allelic balance ratios. The allelic imbalance calling performance is assessed by receiver operating characteristic (ROC) curves. In the absence of copy-number changes, i.e., RAF is 0.5, the performance of the three methods is similar (Fig. 2 a, d, and g).

In the presence of copy-number changes, BaalChIP shows significant improvements in the identification of true ASB events. In Fig. 2, we highlight results on data sets with RAF set to either 0.3 or 0.1, which represent a modest and severe background allelic imbalance due to copy-number changes. Significant improvements can be found across various sequencing depths and across the number of TFs considered. In particular, in the data sets with modest copy-number changes, the performance of BaalChIP is like its performance in the absence of copy-number changes, whereas the performance of both the binomial test and iASseq suffers badly. When RAF is 0.1, BaalChIP is still able to achieve relatively good performance. In addition, the performance of BaalChIP benefits greatly from aggregating binding data for multiple TFs at a SNP (Additional file 1: Figure S3).

Overall, the simulation results show that BaalChIP is robust and performs well across a wide range of variations in the data set. In particular, in regions with copy-number changes, BaalChIP shows significant improvements over the state-of-the-art baselines. Thus, BaalChIP offers a robust analysis of ASB in samples obtained with frequent copy-number changes.

### Case study 1: ENCODE data

We applied BaalChIP to 548 samples from the ENCODE project [19]. In total, 271 ChIP-seq experiments were analyzed, assaying a total of eight cancer and six non-cancer cell lines representing different tissues. The data contained either two or three replicates per experiment and four to 42 DNA-binding proteins per cell line (Additional file 1: Figure S4 and Additional file 4: Table S3 show a summary of the cell lines, tissues, and number of ChIP-seq experiments used in this case study).

The initial number of genotyped heterozygous SNPs per cell line ranged from 139K to 356K. We selected those SNPs that occurred within ChIP-seq peaks in the corresponding cell lines, which amounts to between 1.6% and 5.8% of all SNPs. To ensure we had a reliable set of heterozygous SNPs, we applied the BaalChIP QC step with the default parameters and options. We removed an average of 30.4% of all SNPs that hit peaks (Additional file 1: Figure S5b); 0.8% of the excluded sites were found in regions of problematic read alignments; 7.4% had biases in simulations (Additional file 1: Figure S5a); 16.4% were not consistent between replicates; and 5.8% had only one observable allele (Additional file 1: Figure S5c). After BaalChIP QC, the number of heterozygous SNPs considered for downstream analysis ranged from 1636 to 12,097.

#### Allele-specific copy-number alterations change ChIP-seq read densities

**Fig. 3:**
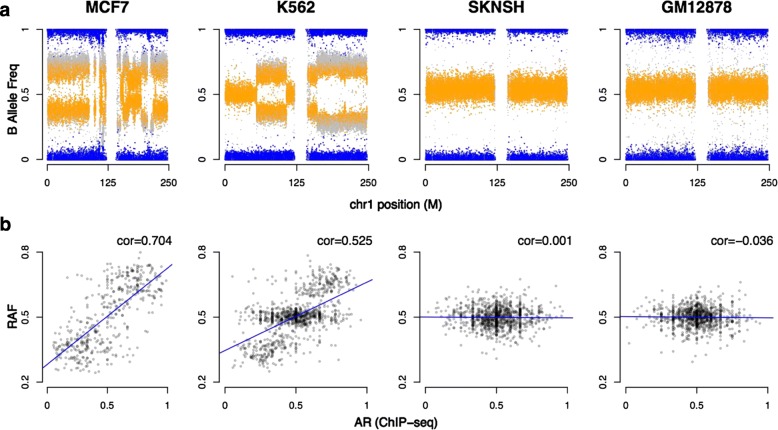
Examples of cancer and non-cancer cell lines from SNP and ChIP-seq ENCODE data. **a** B allele frequencies (BAFs) for chromosome 1 for three cancer cell lines (MCF-7, K562, and SK-N-SH) and one non-cancer cell line (GM12878). Individual SNPs are colored according to genotype values: homozygous AA or BB (*blue*) and heterozygous AB (*orange*). **b** Correlations between the BAF values and the ChIP-seq AR of heterozygous SNPs. RAF corresponds to the BAF value with respect to the reference allele (RAF is equal to BAF if the reference allele corresponds to the B allele; RAF is equal to 1 − BAF if the reference allele corresponds to the A allele). The fitted linear model (*blue line*) and the Spearman correlation coefficient (*cor*) show the relationship between BAF and ChIP-seq ARs at heterozygous sites. *AR* allelic ratio, *BAF* B allele frequency, *chr1* chromosome 1, *cor* correlation, *RAF* reference-allele frequency, *SNP* single-nucleotide polymorphism

First, we examined how allele-specific copy-number alterations affect the ARs observed from ChIP-seq data (Fig. 3). The relative presence of each allele is measured by BAF obtained from SNP arrays [27].

BAF values lie in the interval [ 0,1]. A BAF value of 0.5 indicates the equal presence of the two alleles and is the expected value for heterozygous sites (AB) in a diploid genome, while values of 0 and 1 indicate homozygous genotypes (AA and BB). In a normal non-contaminated diploid sample, a BAF plot will have three bands: one centered at 0.5 for AB genotypes, and two bands at 0 and 1 for the AA and BB homozygous genotypes, respectively.

Figure 3 a presents the BAF plots for chromosome 1 of three cancer cell lines (MCF-7, K-562, and SK-N-SH) and one non-cancer cell line obtained from normal blood (GM12878). As expected, the BAF plot of GM12878 is characterized by a typical diploid pattern of BAF values distributed around 0, 0.5, and 1, corresponding to the diploid genotypes AA, BB, and AB. The few small deviations from these values can be attributed to germ-line-based copy-number aberrations [28, 29]. The BAF plot for the SK-N-SH cell line, a glioblastoma cancer cell line known to have a near diploid karyotype [30], is also relatively stable. In contrast, the MCF-7 and K-562 cancer cell lines show more complex BAF plots due to a variety of copy-number aberrations.

To evaluate the effects of allele-specific copy-number alterations on the ChIP-seq read densities, for all four cell lines we compared the BAF scores with ChIP-seq ARs obtained at heterozygous SNPs (Fig. 3 b). We report ChIP-seq ARs as the proportion of total reads carrying the reference allele. We observed a clear correlation between the BAF scores and the imbalance in the ChIP-seq ARs in the MCF-7 and K-562 cancer cell lines (Spearman *ρ*=0.704 and 0.525, respectively), while this correlation is not observed in the near-diploid SK-N-SH (Spearman *ρ*=0.001) and the normal GM12878 cell lines (Spearman *ρ*=0.036).

The same effect can be observed in all other cell lines (Additional file 1: Figure S6a) and is particularly striking in cancer cells with the highest proportion of extreme BAF scores (<0.4 or >0.6; Additional file 1: Figure S6b). Taken together, these results demonstrate the need to adjust for the background genomic abundance of the alleles when attempting to identify putative cis-acting regulatory SNPs from ChIP-seq data. These data also support our assumption that for allelic changes that do not change TF affinity, the allele frequency in ChIP-seq data is proportional to the presence of these alleles in the gDNA.

#### Allele-specific amplification events explain most of the imbalances observed in ChIP-seq data in cancer cell lines

Having obtained a reliable set of heterozygous SNPs per cell line, we next applied the Bayesian framework implemented in BaalChIP to each of the 14 cell lines individually. To assess the importance of adjusting for copy-number biases, we performed the analysis with and without adjusting for the RAF bias. In a diploid sample, the ARs are expected to be distributed around the 0.5 average, assuming that only a small proportion of sites carry an imbalance towards one of the alleles. However, as shown in Fig. 3, copy-number alterations can affect read densities, which will have an effect on ARs, in particular in cancer cells. Additional file 1: Figure S7a shows the observed AR values in ChIP-seq data compared to the values before and after RAF correction for all analyzed cell lines. We observed that after the RAF copy-number correction, the corrected ARs become more evenly distributed around an average of 0.5. This effect is particularly notable in data obtained from cancer cell lines.

Overall, we found 2438 ASB sites across all cell lines (Additional file 5: Table S4), with an average of 3.1% SNPs displaying ASB using our chosen cutoffs. Additional file 1: Figure S7b demonstrates that the copy-number correction has a strong effect in cancer cell lines. In the most extreme cases, the number of allele-specific imbalances is reduced by fourfold. In normal cells and in cancer cells with near diploid genomes, such as HL-60 and SK-N-SH [30, 31], the total number of identified ASB sites due to copy-number effects is much lower (Additional file 1: Figure S7b). This is consistent with non-cancer cell lines carrying far fewer copy-number aberrations. Because ASB is more easily detected in regions of high sequence coverage due to increases in statistical power (Additional file 1: Figure S8), we repeated the same analysis after selecting only SNPs with 30–40 × read coverage to control for read depth biases and verify the same effect (Additional file 1: Figure S7c). Importantly, the data shown here demonstrate that by adjusting for RAF, we are able to remove artifacts that are caused by copy-number alterations in cancer cell lines.

We observed higher rates of ASB on chromosome X of female cell lines (GM12878, GM12892, IMR90, MCF10, MCF-7, and SK-N-SH) than in autosomal chromosomes (Additional file 1: Figure S9, *χ* ^2^ test *p*<2.2×10^−16^ for chromosome X versus autosomal identified sites, compared for all six cell lines). These cases might be explained by the extent of X inactivation. In normal tissue, X inactivation is random. However, in clonal cell lines, the same X chromosome will continue to be silenced and most X-linked genes are expressed in a mono-allelic fashion [32].

#### ASB events are consistent within and between cell lines

We evaluated the correlation of ARs between pairs of biological replicates, between distinct proteins bound at the same site, and at identical ASB sites in different cell lines. The ARs of all shared heterozygous SNPs are correlated well across biological replicates (Additional file 1: Figure S10). Secondly, different proteins binding to the same SNP also display concordant ARs (Additional file 1: Figure S11) across cell lines. Our observations are consistent with the concordance of ASB of different co-bound TFs described in previous studies [1–3, 10, 12, 33, 34]. The Spearman correlation coefficients of all pairwise comparisons (between replicates and within cells between different DNA-binding proteins at the same SNP) are plotted as a box plot in Additional file 1: Figure S12a. Positive Spearman correlation coefficients are observed in every case, and in the majority of the cases we observe correlation coefficients >0.8. Therefore, overall, we find that BaalChIP generates highly reproducible results, across separate ChIP-seq experiments.

In addition, we analyzed if heterozygous sites shared by cell lines had ARs that were skewed towards the same allele or not. Of the identified ASB sites, only a small proportion (149 out of 2438; 6.1%) were shared between cell lines (Additional file 1: Figure S12b). Of these ASB sites, 91% (136 out of 149; Additional file 6: Table S5) show an agreement in the direction of the allelic bias. Discordant cases may be explained by environmental or epigenetic factors, by the different genomic context in different cells [1, 2], or by the low sequencing coverage of ChIP-seq data. The high proportion of ASB with the same allelic bias further supports the robustness of BaalChIP.

#### Functional annotation of ASB sites

We then examined the genomic distribution of the identified ASB SNPs. A large proportion of ASB SNPs overlap introns and intergenic regions, and a considerable proportion is found at promoter-proximal regions, mainly reflecting the binding patterns of the ChIPed proteins (Additional file 1: Figure S13). A large proportion of ASB SNPs overlap previously predicted enhancer regions and histone modifications associated with active enhancers (H3K4me1 and H3K27ac), with an average of 70.2% of ASB SNPs occurring within cell-type-specific putative enhancer regions (Additional file 1: Figure S14). However, this enrichment is not significant when compared to non-ASB SNPs (Additional file 7: Table S6, *χ* ^2^ *p*>0.05), and it possibly mainly reflects the distribution of the binding sites (ChIP-seq peak regions) from which the initial set of heterozygous SNPs was sampled.

Finally, to examine the putative functional mechanisms of ASB SNPs, we assessed if they modulated TF binding affinity. We used predictions from HaploReg to assess if the observed ASB SNPs alter canonical TF binding motifs. We found that 88% of ASB SNPs (range 83 to 92%) are motif-disrupting SNPs but this enrichment was not statistically significant, since 85% of all tested SNPs under ChIP peaks were also found to alter motif scores. No significant difference in the magnitude of the change in binding affinity was observed between motif-disrupting ASB SNPs when compared to all tested motif-disrupting SNPs using the Kolmogorov–Smirnov test (Additional file 1: Figure S15). To determine which TF motifs are most likely to be disrupted, we grouped SNPs according to the DNA-binding proteins as identified by ChIP-seq peaks, and identified the top motifs disrupted by ASB SNPs compared to non-ASB SNPs. In the majority of cases, the top disrupted motifs match the DNA-binding proteins used to generate the ChIP-seq data (Additional file 8: Table S7). Overall, these results provide good evidence that an ASB we identify represents a true biological phenomenon.

### Case study 2: FAIRE-seq data

To demonstrate the generality of our approach, we applied BaalChIP to targeted FAIRE-sequencing data obtained from two breast-cancer cell lines, MDA-MB-134 and T-47D. FAIRE stands for formaldehyde-assisted isolation of regulatory elements and is an effective method of identifying DNA regions in the genome associated with open chromatin [35]. The method is based on formaldehyde cross-linking being more efficient in nucleosome-bound DNA than it is in nucleosome-depleted regions of the genome. Thus, FAIRE-seq identifies regions of open chromatin. One advantage of FAIRE-seq over ChIP-seq is that the assayed chromatin is not limited to the location of specific DNA-associated proteins.

We chose to focus on the fraction of the genome that has been previously associated with breast-cancer risk. To do so, we selected 75 previously known breast-cancer-risk regions (Additional file 9: Table S8) [36] covering a total of 4.93 Mb of the human genome. We performed targeted sequencing of three replicated FAIRE samples per cell line and the corresponding gDNA controls. Targeted sequencing of the gDNA samples allowed us to determine with confidence the genotype of a high number of sites at the assayed breast-cancer-risk regions. We identified a total of 3208 and 1624 heterozygous SNPs in MDA-MB-134 and T-47D cells, respectively. In this data set, the sequenced gDNA samples were used for the RAF correction step, i.e., ARs at each SNP position were calculated directly from gDNA samples and used for bias correction. We first applied the BaalChIP QC pipeline to eliminate biases. We noticed that none of the SNPs in the selected targeted regions overlapped regions of potential problematic alignments, and only a small proportion of sites were eliminated during the BaalChIP QC step (<0.3%).

**Fig. 4:**
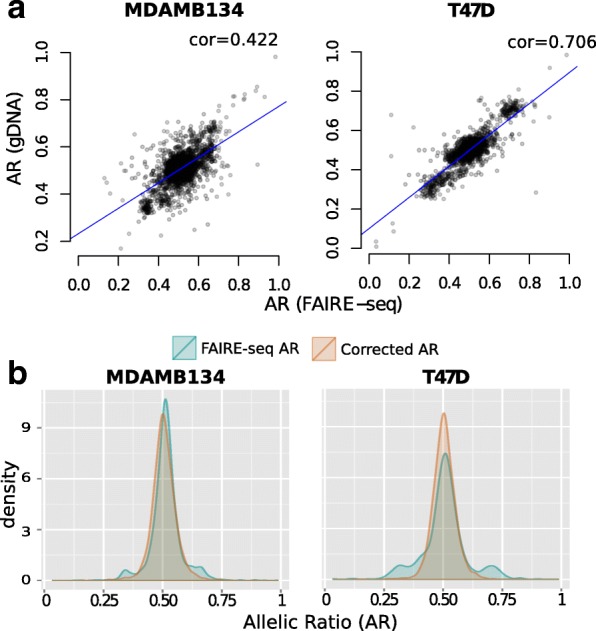
ASB detection from FAIRE targeted sequencing data. **a** Correlations between the allelic ratios obtained from gDNA and FAIRE-seq data. **b** Density plots showing the distribution of allelic ratios before (*green*) and after (*orange*) BaalChIP correction. The adjusted AR values were estimated by the BaalChIP model after taking into account the RAF scores computed directly from the control gDNA samples. *AR* allelic ratio, *ASB* allelic-specific binding, *cor* correlation, *gDNA* genomic DNA, *RAF* reference-allele frequency

We observed a high correlation between ARs in the gDNA and FAIRE samples (Fig. 4 a), indicating that observed allele specificity in FAIRE-seq samples is primarily due to copy-number alterations and must, therefore, be corrected for. Figure 4 b shows the observed ARs obtained for the FAIRE-seq samples compared to the values after correcting for gDNA. After correction, the ARs are more evenly distributed around the average of 0.5. We found that approximately 0.65% (MDA-MB-134) and 0.56% (T-47D) of the tested sites in the selected risk regions were allele-specific. These correspond to a total of 21 sites in MDA-MB-134 and nine sites in T-47D cell lines (Table 1).

**Table 1:** Heterozygous SNPs identified as allele-specific and the correspondent cell lines and alleles in the FAIRE-seq data set

ID	Chrom	Pos	Ref	Alt	ASB allele	Allelic imbalance	Gene name	Location
rs2373062	2	218322137	G	C	T47D-G	0.76	DIRC3	Intron
rs9682063	3	4737842	A	G	T47D-G	0.34	ITPR1	Intron
rs9292122	5	56087910	G	A	T47D-G	0.81		Intergenic
rs201314815	7	144115683	C	T	T47D-T	0.28		Intergenic
rs201949580	7	144115698	T	G	T47D-G	0.30		Intergenic
rs200312388	7	144115704	T	G	T47D-G	0.29		Intergenic
rs200496470	8	128323987	A	G	T47D-G	0.15	CASC8	Intron
rs111929748	11	69379287	G	T	T47D-T	0.32		Intergenic
rs12939887	17	53162672	A	G	T47D-A	0.67	STXBP4	Intron
rs72650670	1	114394681	G	A	MDAMB134-G	0.76	PTPN22	Coding
rs116782319	5	158260122	A	T	MDAMB134-T	0.33	EBF1	Intron
rs12670370	7	144118594	C	A	MDAMB134-C	0.79		Intergenic
rs2392922	8	129166840	A	G	MDAMB134-A	0.79		Intergenic
rs17793170	8	129205679	G	A	MDAMB134-A	0.33		Intergenic
rs2666763	10	22101608	G	A	MDAMB134-A	0.29	DNAJC1	Intron
rs2941733	10	22241656	T	C	MDAMB134-T	0.74	DNAJC1	Intron
rs4752570	10	123337066	T	C	MDAMB134-C	0.32	FGFR2	Intron
rs2912780	10	123337117	C	T	MDAMB134-C	0.69	FGFR2	Intron
rs2912779	10	123337182	T	C	MDAMB134-T	0.72	FGFR2	Intron
rs2981579	10	123337335	A	G	MDAMB134-A	0.71	FGFR2	Intron
rs2912778	10	123338654	G	A	MDAMB134-G	0.73	FGFR2	Intron
rs11200017	10	123339685	G	A	MDAMB134-A	0.26	FGFR2	Intron
rs2981578	10	123340311	C	T	MDAMB134-C	0.88	FGFR2	Intron
rs11599804	10	123340664	G	A	MDAMB134-A	0.16	FGFR2	Intron
rs34032268	10	123341525	A	C	MDAMB134-C	0.19	FGFR2	Intron
rs2936870	10	123348902	T	C	MDAMB134-T	0.69	FGFR2	Intron
rs3135718	10	123353869	C	T	MDAMB134-C	0.67	FGFR2	Intron
rs112536831	19	17424329	G	A	MDAMB134-G	0.75	DDA1	Intron
rs113207944	19	17438923	C	T	MDAMB134-C	0.74	ANO8	Intron
rs10416361	19	44294617	A	G	MDAMB134-A	0.75		Intergenic

Chrom and Pos are the coordinates in the hg19 human reference genome

*Alt* alternative, *ASB* allelic-specific binding, *Ref* reference

Out of the 21 SNPs identified in the MDA-MB-134 cell line, 11 cluster in the 10q26.13 region. This cluster includes the rs2981579 SNP in the second intron of the FGFR2 gene (Table 1), which is the SNP with the strongest association with breast cancer in genome-wide analysis [37]. In the 10q26.13 region, we identify two breast-cancer-risk SNPs, rs2981579 and rs2981578, with a strong allelic imbalance towards their risk alleles, rs2981579-A and rs2981578-C, respectively.

### Comparing BaalChIP to competing methods

We applied two competing methods, the binomial test and the iASeq [10] method, to the ENCODE and FAIRE-seq data sets.

When applying the iASeq method to the ENCODE data set, the existence of missing data created an error. Missing data occur for those samples that do not contain ChIP-seq peaks at the SNP in question, or if the SNP did not pass the previously applied QC step for that sample. To overcome this limitation of iASeq, we replaced every missing data point by zero, with the caveat that this ad hoc approach may create unknown biases in our results.

**Fig. 5:**
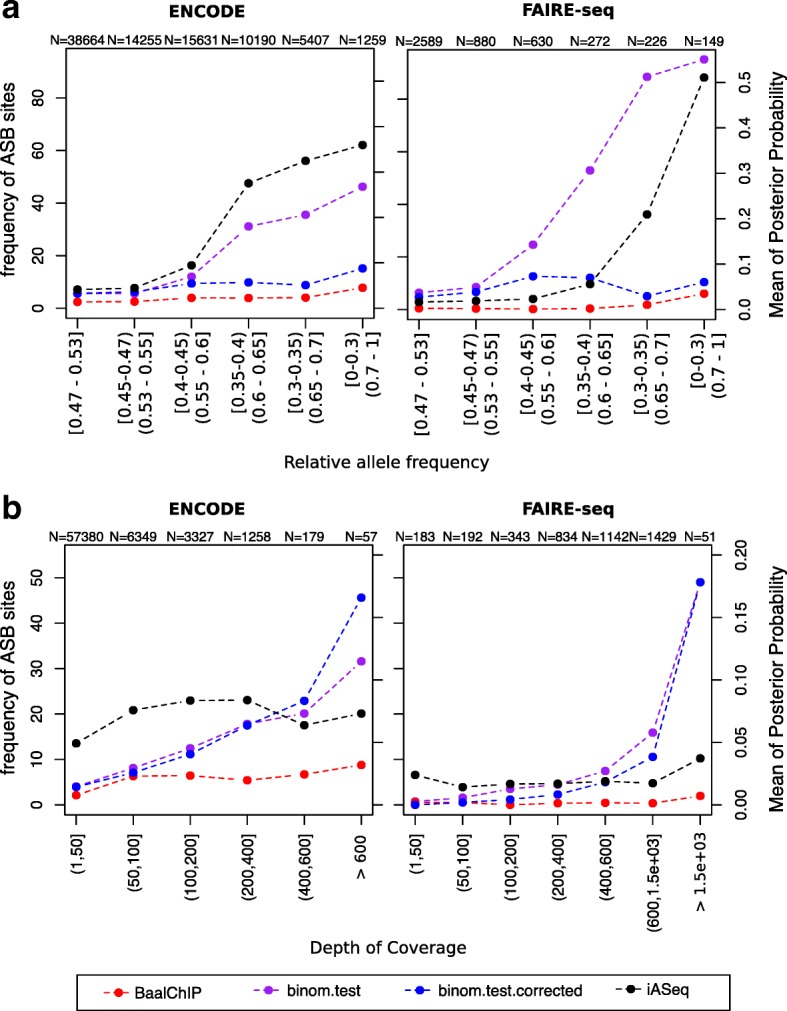
Comparison of BaalChIP with other available methods. Left *y*-axis corresponds to the frequency of ASB events called by BaalChIP or the binomial tests (*red, blue and purple lines*). Right *y*-axis corresponds to the mean of the posterior probability given by the iASeq method (*black line*). The numbers at the top of the plot show the total number of tested heterozygous sites in each bin. **a** SNPs were grouped in bins of different RAF intervals. The RAF intervals increase in terms of distance to the diploid value (RAF = 0.5). The binomial test (without RAF correction; *purple line*) and the iASeq methods (*black line*) are biased towards the detection of ASB events in regions of altered copy numbers. **b** SNPs were grouped in bins of different depth of coverage. SNPs in regions RAF < 0.4 or RAF > 0.6 were excluded from this analysis. When applying the binomial test (*purple* and *blue lines*), the frequency of ASB detection increases for higher covered sites, while the same is not true when applying BaalChIP or the iASeq methods. The effect is particularly visible for the FAIRE-seq data set. *ASB* allelic-specific binding, *binom* binomial, *RAF* reference-allele frequency, *SNP* single-nucleotide polymorphism

When applying the binomial test, for each cell line, we pooled read count data from different experiments and replicates. While this approach maximizes statistical power, it may mask heterogeneity in ASB obtained from different experiments. Previous studies have shown that at least 20 × read coverage at a particular SNP position is necessary to reach adequate statistical power with the binomial test [9, 12]. Therefore, we restricted our analysis to SNPs with a coverage of at least 20 reads in the pooled data. We included two different ways of performing the binomial test, either by setting the probability of success to 0.5 or by weighting the binomial null with the RAF scores. In real data, unlike simulations, we are not able to access the true-positive and false-positive rates and compute ROC curves. For this reason, we mainly focused on testing whether BaalChIP is comparable to existing methods while improving the biased ASB detection in high copy-number regions (Fig. 5 a) or the problem of overdispersion in the data (Fig. 5 b).

First, for consistency of the analysis, we obtained a set of heterozygous SNPs to test using the BaalChIP QC step. We then compared BaalChIP to the binomial test method with (*p*= RAF) and without (*p*=0.5) copy-number correction based on how many of the identified ASB SNPs were in Copy number alterations (CNA) regions. For the comparison with the iASeq method, we computed the estimated posterior probability obtained for SNPs for different regions of altered copy numbers. Figure 5 a shows that the binomial test (without RAF correction) and the iASeq method show a substantial bias towards the increased detection of ASB events in regions of altered copy number, as expected.

Both BaalChIP and iASeq employ a beta-binomial distribution to model read count data, which accounts for extra-binomial variability (overdispersion). To check for the effect of overdispersion in the ENCODE and FAIRE-seq data, we considered only SNPs at regions with normal or close to normal copy numbers (RAF between 0.4 and 0.6) and computed the percentage of detected ASB sites in bins of increasing depth of coverage. Overdispersion is often more accentuated for sites of higher coverage (>200 pooled total counts), which creates a particularly sensitive scenario for the detection of ASB sites using the binomial distribution. Figure 5 b demonstrates clearly the effect of overdispersion in the FAIRE-seq data, particularly visible at sites of high coverage, which shows the advantage of BaalChIP and iASeq over the binomial model. This effect is less pronounced in the ENCODE data, where the depth of coverage is overall lower (Additional file 1: Figure S16).

These results suggest that for higher read counts, random fluctuations in ARs should be modeled with a beta-binomial distribution, rather than a binomial distribution, to reduce the number of false-positive results.

## Discussion

ASB analysis is an important method for identifying putative regulatory SNPs that might have an effect on TF binding and gene expression and might be associated with disease phenotypes. Our method for the Bayesian analysis of allelic imbalances from ChIP-seq data, called BaalChIP, has been motivated by the need to address the issue of detecting allelic-specific imbalances from ChIP-seq data obtained specifically from cancer cell lines, which frequently carry copy-number alterations.

While allele-specific copy-number alterations can be associated with changes in transcript abundance [38] and are possibly implicated in cancer phenotypes, they can confound the identification of ASB at potential regulatory sites. Traditional methods of identifying ASB have not been able to distinguish between effects that are caused by allele-specific amplification of binding sites versus the effects caused by ASB by specific TFs. Our method now allows us to distinguish these and will, therefore, help us to delineate better the causal mechanisms of disease.

BaalChIP is a general framework applicable to a wide range of assays. We have applied it to ENCODE ChIP-seq and our own FAIRE-seq samples, and demonstrated the utility of BaalChIP to identify ASB events from cancer genomes. Each of these chromatin assays has its own advantages in the detection of ASB. ChIP-seq precisely determines the location of specific DNA-associated proteins, while FAIRE-seq identifies broader regions of open chromatin, which might be less informative in terms of pinpointing the functional regulatory elements of the genome. On the other hand, the ChIP assay is limited to the availability of high-quality antibodies and only one factor can be tested per experiment. It is still an open question which chromatin assay will be the most informative for understanding ASB differences.

Cancer cell lines carry frequent copy-number alterations. Therefore, an important issue is the determination of the background biases in allele frequency observed in regions of altered copy number. For the ENCODE data set, we have demonstrated that BAF scores obtained from microarrays could be used to correct for these effects in the ARs; however, it is also noteworthy that the microarray technology used by the ENCODE study did not generate BAF scores comprehensively for all possible heterozygous sites, limiting the number of SNPs at which the allele specificity could be assayed. Therefore, for the majority of the samples in the ENCODE case study, it is likely that the regulatory potential of true causal SNPs was not directly assayed. As demonstrated by the analysis of FAIRE-seq data, this issue can be overcome by including gDNA-sequencing as control samples to detect allele-specific biases, provided that there is sufficient read coverage at each site.

The identified ASB sites may form the basis for future functional analysis of the genome. Of particular interest, within the FGFR2 gene, rs2981578 has been previously suggested to be the key causal variant. Indeed, this SNP displays the highest allelic imbalance in a breast-cancer cell line (Table 1).

BaalChIP has been designed for data from cell lines, not tumor samples [39]. Normal contamination and the existence of different clonal subpopulations in tumor samples pose additional challenges and can distort the expected distribution of heterozygous variant allele fractions. In the future, these challenges can potentially be overcome by combining the ideas implemented in BaalChIP with probabilistic methods developed for dissecting genetic heterogeneity and cancer evolution [40].

## Conclusions

In summary, BaalChIP is a rigorous probabilistic method to detect allelic imbalance by correcting for the effect of background allele frequency on observed ChIP-seq read counts. BaalChIP implements stringent filtering and preprocessing steps and allows the joint analysis of multiple ChIP-seq samples across a single variant. In simulations, BaalChIP outperformed competing approaches, and when applied to 548 ENCODE ChIP-seq and six targeted FAIRE-seq samples, BaalChIP effectively corrected the allele-specific analysis for copy-number changes and increased the power to detect putative cis-acting regulatory variants in cancer genomes.

## Methods

### BaalChIP quality control and filtering

The QC and filtering steps implemented in the BaalChIP R package are:

1. Keep only reads with mapping quality >15 and with base quality >10.
2. Within each cell line, consider only heterozygous SNPs overlapping TF binding sites (TFBSs), identified by ChIP-seq peak calling.
3. Exclude sites susceptible to allelic mapping bias in regions of known problematic read alignment [22–24].
4. Apply simulation-based filtering to exclude SNPs with intrinsic bias to one of the alleles [6, 17].
5. Consider only SNPs that are represented in all replicated samples after applying all previous filters.
6. Exclude possibly homozygous SNPs where only one allele was observed after pooling ChIP-seq reads from all examined samples [4, 26].

#### Generating allele counts per SNP

For each SNP, the number of reads carrying the reference and alternative alleles are computed using the pileup function and the PileupParam constructor of the Rsamtools package [41]. For each BAM file, BaalChIP considers only heterozygous SNPs overlapping the genomic regions in the corresponding BED files (peaks). Two arguments of the PileupParam constructor can be manipulated by the user: min_mapq, which refers to the minimum mapping quality for an alignment to be included in pileup (reads with a mapping quality lower than this threshold are ignored; default is 15), and min_base_quality, which refers to the minimum quality for each nucleotide in an alignment (bases at a particular location with base quality lower than this threshold are ignored; default is 10).

#### Data sources for regions of problematic alignments

By default, BaalChIP considers three sets of regions known to be of problematic read alignment: (1) blacklisted regions downloaded from the UCSC Genome Browser (mappability track; hg19, wgEncodeDacMapabilityConsensusExcludable and wgEncodeDukeMapabilityRegionsExcludable tables), (2) non-unique regions selected from the DUKE uniqueness mappability track of the UCSC genome browser (hg19, wgEncodeCrgMapabilityAlign50mer table), and (3) collapsed repeat regions downloaded from [24] at the 0.1% threshold. Sets of filtering regions used in this filtering step are fully customized and additional sets can be added by the user as GenomicRanges objects [42].

#### Simulations to identify SNPs with intrinsic biases

For each heterozygous site, BaalChIP simulates every possible read overlapping the site in four possible combinations: reads carrying the reference allele (plus and minus strands) and reads carrying the alternative allele (plus and minus strands). The simulated reads are constructed based on published methodology (scripts can be shared by Degner et al. upon request) [6] without taking into consideration different qualities at each base in the read or different read depths of coverage. As described by Degner et al., these parameters were sufficient to predict the SNPs that show an inherent bias [6].

Reads are then aligned to the reference genome and BAM files are generated using Bowtie version 1.1.1 [43] and Picard Tool version 1.47. The pipeline used to generate and align simulated reads can be fully customized with other aligners (e.g., BWA) and it is available under the file name run_simulations.sh found in the folder extra of the BaalChIP R package. Simulated allelic read counts are computed using the pileup function and the PileupParam constructor of the Rsamtools package [41]. For each SNP, the correct number of reads that should map to the reference and non-reference alleles is known (it corresponds to twice the read length for each allele). Sites with an incorrect number of read alignments are discarded from the analysis.

#### Estimating reference mapping bias

The reference mapping bias is calculated as described in [4, 26]. First, reads are combined across all heterozygous sites that pass the previous QC steps. The expected AR (reference/total) for each cell line is then calculated separately for each allele combination. A minimum of 200 sites is required for each category. If there was less, a global estimate was used for that category. For each SNP, this computation results in an AR *μ*∈(0,1). These ARs are used as part of a prior distribution in the Bayesian model described in the next section.

### BaalChIP Bayesian model description

BaalChIP is designed to infer an allelic imbalance from read counts while integrating copy-number and technical bias information. We assume that we are given a set of *N* data sets covering a SNP position. We jointly infer the allelic imbalance from signals in all data sets covering the same SNP.

For the *n*th data set, let $d_{n} \in \mathbb {N}$ denote the total number of reads covering a SNP position and $a_{n} \in \mathbb {N}$ the number of reads reporting the reference allele. BaalChIP models *a*_*n*_ with a beta-binomial distribution, i.e., the binomial distribution in which the probability of success is integrated out given that it follows the beta distribution:

1 $$\begin{array}{*{20}l} p(a_{n}\,\,|\,\,d_{n}, \alpha, \beta) = \operatorname{BetaBin}(a_{n}\,\,|\,\,d_{n}, \alpha, \beta). \end{array} $$

The beta-binomial distribution controls for overdispersion, i.e., that the increased variance in next-generation sequencing data cannot be captured in the standard binomial model [18]. To gain a more intuitive interpretation of the parameters, we re-parameterize *α* and *β* in terms of the precision of the beta-binomial distribution, *Λ*, and the mean probability that a reference read is observed, *θ*:

2 $$\begin{array}{*{20}l} \alpha &= \theta\Lambda, \end{array} $$

3 $$\begin{array}{*{20}l} \beta &= (1-\theta)\Lambda. \end{array} $$

#### Including reference mapping bias

In the next level of our model, we place a beta distribution over *θ*, which allows us to include naturally the technical reference mapping bias:

4 $$\begin{array}{*{20}l} p(\theta\,\,|\,\,\alpha_{0},\beta_{0}) = \text{Beta}\left({\theta}\,\,|\,\,\alpha_{0},\beta_{0} \right), \end{array} $$

where, as before, we re-parameterize the shape parameters *α*_0_ and *β*_0_ by the mean and variance of the distribution. Denoting the variance by *λ* and using as the mean the reference mapping bias *μ*∈(0,1), as discussed above, we get:

5 $$\begin{array}{*{20}l} \alpha_{0} &= \left(\frac{(1-\mu)}{\lambda} - \frac{1}{\mu}\right) \mu^{2}, \end{array} $$

6 $$\begin{array}{*{20}l} \beta_{0} &=\left(\frac{(1-\mu)}{\lambda} - \frac{1}{\mu} \right) \left(\mu - \mu^{2}\right). \end{array} $$

As a consequence of this re-parameterization, the reference mapping bias has a very intuitive interpretation as the a priori mean probability that a read reporting the reference allele is observed.

#### Including reference allele frequency

Next, we re-parameterize *θ* as a function of the allelic balance ratio *η* and reference allele frequency *ρ*:

7 $$\begin{array}{*{20}l} \theta \equiv f(\eta, \rho) = \frac{\eta\rho}{\eta\rho+ (1-\eta)(1-\rho)}. \end{array} $$

Intuitively, this parameterization implements Bayes’s rule with the following definitions:

$$\begin{array}{*{20}l} \theta &= \text{Prob}(\text{allele}=\text{Ref}\ |\ \text{binding} = \text{Yes}),\\ \eta &= \text{Prob}(\text{binding}=\text{Yes}\ |\ \text{allele} = \text{Ref}), \\ 1-\eta &\approx \text{Prob}(\text{binding}=\text{Yes}\ | \ \text{allele}=\text{Alt}), \\ \ \rho &= \text{Prob}(\text{allele}=\text{Ref}),\\ \ 1-\rho &= \text{Prob}(\text{allele}=\text{Alt}). \end{array} $$

The auxiliary condition on binding status in the definition of *θ* reflects that we analyzed only peaks and, thus, it assumes that observed ChIP-seq reads are obtained after TF binding. Equation 7 then formulates a ChIP-seq experiment as a process to obtain the posterior probability of a TF binding to the reference allele. In this model, the allelic balance ratio acts as the likelihood for TF binding to the reference allele and the RAF as the prior.

To understand better how this model works, we will discuss two illustrative scenarios. Example 1: Assuming no copy-number alterations, i.e., RAF *ρ*=0.5, and no allelic imbalance, i.e., *η*=0.5, then

$$\theta = \frac{0.5\times0.5}{0.5\times0.5 + 0.5\times0.5} = 0.5, $$

which shows that BaalChIP contains the assumption of the previous approaches as a special case. Example 2: In the event of Loss of heterozygosity (LOH) on the alternative allele, RAF *ρ*=1, then irrespective of the allelic balance ratio, all observed reads will report the reference allele, which is reflected by

$$\theta=\frac{\eta \times 1 }{\eta \times 1 + (1-\eta) \times 0} = 1. $$

#### Posterior distribution of allelic imbalance

To represent the uncertainty in the data rigorously, we adopt a full Bayesian approach targeting the posterior distribution of the allelic balance ratio *η*. Since the change of variables in Eq. 7 and the prior on *θ* directly define a prior over *η*, namely

8 $$ p(\eta|\rho, \mu, \lambda) = p(f(\eta, \rho)|\mu, \lambda)\frac{\partial f(\eta, \rho)}{ \partial \eta},  $$

we can write the posterior of *η* as follows:

9 $$\begin{array}{*{20}l} &{}p\left(\eta\,|\,\{a_{n}\}_{1}^{N}, \{ b_{n} \}_{1}^{N}, \rho, \Lambda, \mu, \lambda \right) \\  &{} \qquad \propto \prod_{n=1}^{N} p(a_{n}|d_{n},f(\!\eta, \rho), \Lambda)\ \!p(f(\eta, \rho)|\mu, \lambda)\ \frac{\partial f(\eta, \rho) }{\partial \eta}, \end{array} $$

where

10 $${} \frac{\partial f(\eta,\rho)}{\partial \eta} =\! \frac{\rho}{\eta \rho + (1-\eta)(1-\rho)} - \frac{\eta \rho (2 \rho - 1)} {(\eta \rho + (1-\eta)(1-\rho))^{2}}.  $$

#### Inference of allelic imbalance

We use the Metropolis–Hastings algorithm with the random walk proposal to approach this distribution. In practice, we fix *Λ*=1000 and *λ*=0.05, which in our experience results in robust performance across a wide range of simulated and real data sets. Allelic imbalance calling is based on the highest posterior density interval, which is constructed from the Markov chain Monte Carlo trace of *η* as the shortest interval containing 95*%* of the sampled values. An allelic imbalance is called if the highest posterior density does not contain the value 0.5, which is a rigorous way to decide that the data cannot be explained well by balanced alleles.

### In silico validations

To thoroughly test the performance of the allelic imbalance calling performance of BaalChIP, read count data are generated using a wide range of parameter settings. Specifically, we varied the number *N* of data sets from 1 to 45, *ρ* from 0.1 to 0.9 in steps of size 0.1, and *d* from 1 to 100 in steps of size 7. Then, 1000 values of *η* were evenly sampled from 0.1 to 0.9. These settings result in a set of 6,075,000 SNPs. The detailed simulation protocol is as follows.

For each *n*∈ [ 1,…,*N*], *ρ*∈ [ 0.1,…,0.9] and *η*∈[ 0.1,…,0.9]:

1. Draw the number of available reference alleles:
  $$t_{n} \sim \text{Binomial}(d_{n}, \rho). $$
2. Draw the read counts reporting a reference allele:
  $$\begin{array}{*{20}l} & \text{If}\ \eta \leq 0.5\text{:} \\ & \quad a_{n} \sim \text{Binomial}(t_{n}, 2\eta). \\ & \text{If}\ \eta > 0.5\text{:} \\ & \quad b_{n} \sim \text{Binomial}(d_{n} - t_{n}, 2(1-\eta)), \\ & \quad a_{n} = d_{n} - b_{n}. \end{array} $$

The performance is measured by ROC curves. The true allelic imbalance is determined if the true *η* is outside the interval [ 0.45,0.55].

### Baseline methods

We compared BaalChIP against two baseline approaches: the binomial test and iASeq [10]. Two-sided binomial tests are performed with the R function binom.test. To correct for reference mapping bias, the null hypothesis on the probability of success is set to be the previously estimated reference mapping bias (RAF), instead of 0.5. We pooled data from all ChIP-seq samples for each analyzed cell line to maximize power. In the analysis of real data, binomial test *p* values were corrected for multiple testing using the false discovery rate (FDR) threshold of 0.01. FDR was calculated with the p.adjust function in R. For the simulated data, the ROC curves for the binomial test are constructed based on the distance between the reference mapping bias and the mean of the 95*%* confidence intervals for the probability of success. All iASseq analyses are performed with the function iASeqmotif where the number of non-null motifs is set to be a vector [ 1,2,…,5], the maximum number of iterations is 300, and the tolerance level of error is 0.001. The ROC curves for iASseq are constructed with bestmotif$p.post, which is the posterior probability for each SNP being an allele-specific event.

### Cell culture

MDA-MB-134 and T-47D human breast cancer cells were cultured in Roswell Park Memorial Institute medium (RPMI) (Invitrogen) supplemented with 10% fetal bovine serum and antibiotics. All cells were maintained at 37 °C, 5% CO_2_. All cell lines were from the CRUK Cambridge Institute biorepository collection. Cell lines were authenticated by short tandem repeat genotyping using the GenePrint 10 (Promega) system and confirmed to be mycoplasma free.

### FAIRE and gDNA purification

FAIRE was performed as previously described [35] with minor adaptations. Briefly, cells were fixed for 10 mins in 1% formaldehyde in fetal calf serum-free medium, washed, and frozen. Nuclei from MDA-MB-134 (3×10^7^ cells/tube) and T-47D (1.5×10^7^ cells/tube) were isolated and sonicated using 300 µl volumes in 1.5 ml Eppendorfs, using a Diagenode Bioruptor. Sonication was performed for 20 cycles of 30 s on/off at the high setting. The sonicate was subjected to three consecutive phenol–chloroform–isoamyl alcohol (25:24:1) extractions and reverse cross-linked overnight. DNA was purified by ethanol precipitation and quantified by Quanti-iT. gDNA was isolated using a Qiagen DNeasy Blood and Tissue Kit according to the protocol.

### Agilent SureSelect amplification, library preparation, and sequencing

DNA fragments were prepared for sequencing using the recommended protocol for SureSelect-Illumina sequencing. Altogether, 69 known breast-cancer-risk-tagging SNPs were retrieved from [36]. The SNAP Proxy Search was used to find SNPs correlated with the 69 tagging SNPs (*r*2>0.6 for 59 of the risk loci and *r*2>0.8 for eight of the risk loci, within a distance of 500 bp) using 1000 Genomes pilot 1 data. Genomic intervals were defined by the leftmost to the rightmost SNP in each Linkage disequilibrium (LD) block with an additional 400 bp of flanking regions. An additional set of seven SNPs with 500 bp of flanking sequences were added manually to include the CCND1 (rs75915166 and rs494406), MAP3K1 (rs10461612, rs112497245, and rs17432750) and TERT gene regions (rs2736107 and rs7705526). In total, we targeted 69 non-overlapping regions comprising 4.93 Mb using the SureSelect method (Additional file 9: Table S8). DNA obtained from the SureSelect solution-based sequence capture was subjected to Illumina HiSeq paired-end sequencing (Illumina). Paired-end sequencing was performed according to the manufacturer’s protocols.

### Preprocessing ENCODE samples

We used publicly available ENCODE ChIP-seq and genotype data sets for a total of 548 samples representing 271 different experiments. We included eight cancer and six non-cancer cell lines representing different tissues. The accession numbers of all public ChIP-seq data sets used in this study are provided in Additional file 3: Table S2. Additional file 4: Table S3 is a summary of all tissues, cell lines, and the number of experiments included in this study. The ChIP-seq data were downloaded as reads mapped to the hg19 genome (BAM files) and corresponding peak calling files (BED files). Duplicated reads were marked using Picard Tool version 1.47. ChIP-seq peak files were merged between replicates using mergePeaks of HOMER version 5.4 with the default option -d to consider only peak ranges that overlapped for all replicates. Heterozygous SNPs and BAF tracks were retrieved from the UCSC Genome browser (hg19, wgEncodeHaibGenotype track, wgEncodeHaibGenotypeBalleleSnp2015-03-04.tsv, and wgEncodeHaibGenotypeGtypeSnp2014-09-15.tsv files). The initial number of genotyped SNPs in the ENCODE files is 1.2 million. We considered only SNPs listed in dbSNP (version 137 [44]). Homozygous SNPs (i.e., BAF > 0.9 or < 0.1) and SNPs with missing BAF scores were removed from the BAF tracks. BAF scores were converted to RAF scores using the information about A and B alleles in the manifest file for the Illumina Human1M-Duo BeadChip (v3.0) downloaded from http://support.illumina.com/array/array_kits/human1m-duo_dna_analysis_kit/downloads.html.

### Preprocessing FAIRE-seq samples

For sequence data from all FAIRE-seq and control samples, sequences were aligned to the human reference genome (GRCh37) using BWA version 0.7.12 [45]. Duplicates were removed using Picard Tool version 1.47 and overlapping reads were clipped using the clipOverlap tool from the bamUtil repository version 1.0.14 with default parameters. SNPs were identified from gDNA samples using the Genome Analysis ToolKit 3.4-46 [46] across all gDNA samples simultaneously. As per GATK Best Practices recommendations [47, 48], duplicated reads were removed and local realignment and base quality recalibration were employed prior to SNP calling. Called SNPs were filtered using a hard filtering criteria (QualByDepth (QD) < 2.0, FisherStrand (FS) > 60.0, RMSMappingQuality (MQ) < 30.0, MappingQualityRankSumTest (MQRankSum) < −12.5, ReadPosRankSumTest (ReadPosRankSum) < −8.0).

### Applying BaalChIP to ENCODE and FAIRE-seq samples

To ensure that we had a reliable set of heterozygous SNPs, we applied the BaalChIP (version 0.1.9) quality control step and considered only uniquely mapping reads with mapping quality > 15 and base call quality > 10. For the ENCODE data set, we removed from the analysis sets of SNPs based on the default six QC filters implemented within the BaalChIP pipeline. The FAIRE-seq samples contained paired-end sequenced reads of 125 bp. Since longer and paired-end reads reduce the uncertainty of read alignment, we did not consider two of the QC filters that are more relevant for shorter read lengths (of less than 50 bp): the unique mappability and intrinsic bias filters. BaalChIP (version 0.1.9) ASB Bayesian analysis was performed with the default parameters and options [21].

### Consistency of ARs

Consistency of the ARs observed at ASB SNPs was analyzed (i) between replicates: pairs of replicated samples, (ii) within cell lines: pairs of ChIP-seq data sets from different proteins (pooled replicate data) in the same cell line, and (iii) between cell lines: pairs of cells (pooled data for each cell line). Correlations were calculated with the Spearman correlation and required at least 15 shared ASB sites.

### SNP annotations relative to genes

To annotate ASB SNPs with respect to gene annotations (5 ^′^ untranslated region, 3 ^′^ untranslated region, promoter, splice site, coding, intron, or intergenic), we used the VariantAnnotation package (version 1.8.13) in R. To obtain the list of known genes and coordinates, we used known gene annotations from the UCSC genome browser obtained from the TxDb.Hsapiens.UCSC.hg19.knownGene library (version 2.6.2) in R [20].

### Overlap with predicted enhancers

To determine if ASB SNPs were enriched in any of the putative enhancer regions, we calculated the overlap of intergenic ASB SNPs in putative enhancer regions. The significance of an observed overlap was determined by a *χ* ^2^ test by comparing the fraction of ASB SNPs in putative enhancer regions with the fraction of non-ASB heterozygous SNPs in putative enhancer regions. Putative enhancer regions were retrieved for six cell lines (GM12878, H1hESC, HeLa, HepG2, K562, and A549) based on predicted weak and strong enhancer sites and/or H3K27ac and H3K4me1 chromatin marks. Enhancer site predictions were retrieved from human segmentations previously generated based on ENCODE data using Segway (downloaded from the wgEncodeAwgSegmentation UCSC genome browser track).

### TF motif disruption analysis

To annotate the potential regulatory effects of the tested SNPs on TFBS motifs, the publicly available HaploReg v2 database (accessible at http://www.broadinstitute.org/mammals/haploreg/haploreg_v2.php) was used. HaploReg calculates allele-specific changes in the log-odds (LOD) scores for the position weight matrices of a regulatory motif based on a library of position weight matrices constructed from TRANSFAC, JASPAR, and protein-binding microarray experiments [49]. The magnitude of the change in binding affinity was calculated as the absolute difference (delta) of LOD scores (delta = LOD(ref) − LOD(alt)). The Kolmogorov–Smirnov test was used to compare the distributions of the absolute delta scores of motif-disrupting ASB SNPs and all tested motif-disrupting SNPs. To determine which TFBS motifs that ASB SNPs were more likely to disrupt, SNPs were grouped according to DNA-binding proteins as identified by ChIP-seq peaks. For each group, a Fisher exact test was used to identify TFBS motifs that were more significantly disrupted by ASB SNPs compared to non-ASB SNPs.

## Additional files

Additional file 1
**Supplementary Figures S1–S16.** (PDF 6500 kb)

Additional file 2
**Supplementary Table S1.** (XLS 24 kb)

Additional file 3
**Supplementary Table S2.** (XLSX 83 kb)

Additional file 4
**Supplementary Table S3.** (XLSX 40 kb)

Additional file 5
**Supplementary Table S4.** (XLSX 177 kb)

Additional file 6
**Supplementary Table S5.** (XLSX 53 kb)

Additional file 7
**Supplementary Table S6.** (XLSX 46 kb)

Additional file 8
**Supplementary Table S7.** (XLSX 50 kb)

Additional file 9
**Supplementary Table S8.** (XLSX 53 kb)

## References

[1] McDaniell R, Lee BK, Song L, Liu Z, Boyle AP, Erdos MR (2010). Heritable individual-specific and allele-specific chromatin signatures in humans. Science.

[2] Reddy TE, Gertz J, Pauli F, Kucera KS, Varley KE, Newberry KM (2012). Effects of sequence variation on differential allelic transcription factor occupancy and gene expression. Genome Res.

[3] Kasowski M, Kyriazopoulou-Panagiotopoulou S, Grubert F, Zaugg JB, Kundaje A, Liu Y (2013). Extensive variation in chromatin states across humans. Science.

[4] Kilpinen H, Waszak SM, Gschwind AR, Raghav SK, Witwicki RM, Orioli A (2013). Coordinated effects of sequence variation on DNA binding, chromatin structure, and transcription. Science.

[5] McVicker G, van de Geijn B, Degner JF, Cain CE, Banovich NE, Raj A (2013). Identification of genetic variants that affect histone modifications in human cells. Science.

[6] Degner JF, Marioni JC, Pai AA, Pickrell JK, Nkadori E, Gilad Y (2009). Effect of read-mapping biases on detecting allele-specific expression from RNA-sequencing data. Bioinformatics.

[7] Rozowsky J, Abyzov A, Wang J, Alves P, Raha D, Harmanci A (2011). Alleleseq: analysis of allele-specific expression and binding in a network framework. Mol Syst Biol.

[8] Satya RV, Zavaljevski N, Reifman J (2012). A new strategy to reduce allelic bias in RNA-Seq readmapping. Nucleic Acids Res.

[9] Skelly DA, Johansson M, Madeoy J, Wakefield J, Akey JM (2011). A powerful and flexible statistical framework for testing hypotheses of allele-specific gene expression from RNA-Seq data. Genome Res.

[10] Wei Y, Li X, Wang Q-F, Ji H (2012). iASeq: integrative analysis of allele-specificity of protein-DNA interactions in multiple ChIP-seq datasets. BMC Genomics.

[11] Mayba O, Gilbert HN, Liu J, Haverty PM, Jhunjhunwala S, Jiang Z (2014). MBASED: allele-specific expression detection in cancer tissues and cell lines. Genome Biol.

[12] Li G, Bahn JH, Lee JH, Peng G, Chen Z, Nelson SF (2012). Identification of allele-specific alternative mRNA processing via transcriptome sequencing. Nucleic Acids Res.

[13] Chen J, Rozowsky J, Galeev TR, Harmanci A, Kitchen R, Bedford J (2016). A uniform survey of allele-specific binding and expression over 1000-genomes-project individuals. Nat Commun.

[14] Almlöf JC, Lundmark P, Lundmark A, Ge B, Pastinen T, Goodall AH (2014). Single nucleotide polymorphisms with cis-regulatory effects on long non-coding transcripts in human primary monocytes. PLoS ONE.

[15] Bailey SD, Virtanen C, Haibe-Kains B, Lupien M (2015). ABC: a tool to identify SVNs causing allele-specific transcription factor binding from ChIP-seq experiments. Bioinformatics.

[16] Liu Z, Gui T, Wang Z, Li H, Fu Y, Dong X (2016). cisASE: a likelihood-based method for detecting putative cis-regulated allele-specific expression in RNA sequencing data. Bioinformatics.

[17] Pickrell JK, Marioni JC, Pai AA, Degner JF, Engelhardt BE, Nkadori E (2010). Understanding mechanisms underlying human gene expression variation with RNA sequencing. Nature.

[18] Roth A, Khattra J, Yap D, Wan A, Laks E, Biele J (2014). PyClone: statistical inference of clonal population structure in cancer. Nat Methods.

[19] ENCODE Project Consortium (2012). An integrated encyclopedia of DNA elements in the human genome. Nature.

[20] R Core Team. R: a language and environment for statistical computing; 2014. http://www.R-project.org/.

[21] de Santiago I, Liu W, O’Reilly M, Yuang K, Chilamakuri SRC, Ponder BAJ, et al. BaalChIP: Bayesian analysis of allele-specific transcription factor binding in cancer genomes. R package version 1.0.0. 2016. https://bioconductor.org/packages/release/bioc/html/BaalChIP.html. 10.1186/s13059-017-1165-7 PMC5326502 28235418

[22] Castel SE, Levy-Moonshine A, Mohammadi P, Banks E, Lappalainen T (2015). Tools and best practices for data processing in allelic expression analysis. Genome Biol.

[23] Fujita PA, Rhead B, Zweig AS, Hinrichs AS, Karolchik D, Cline MS (2011). The UCSC genome browser database: update 2011. Nucleic Acids Res.

[24] Pickrell JK, Gaffney DJ, Gilad Y, Pritchard JK (2011). False positive peaks in ChIP-seq and other sequencing-based functional assays caused by unannotated high copy number regions. Bioinformatics.

[25] Carroll TS, Liang Z, Salama R, Stark R, de Santiago I (2014). Impact of artifact removal on chip quality metrics in ChIP-seq and ChIP-exo data. Front Genet.

[26] Lappalainen T, Sammeth M, Friedländer MR, AC‘t Hoen P, Monlong J, Rivas MA (2013). Transcriptome and genome sequencing uncovers functional variation in humans. Nature.

[27] Peiffer DA, Le JM, Steemers FJ, Chang W, Jenniges T, Garcia F (2006). High-resolution genomic profiling of chromosomal aberrations using Infinium whole-genome genotyping. Genome Res.

[28] Iafrate AJ, Feuk L, Rivera MN, Listewnik ML, Donahoe PK, Qi Y (2004). Detection of large-scale variation in the human genome. Nat Genet.

[29] Sebat J, Lakshmi B, Troge J, Alexander J, Young J, Lundin P (2004). Large-scale copy number polymorphism in the human genome. Science.

[30] Biedler JL, Helson L, Spengler BA (1973). Morphology and growth, tumorigenicity, and cytogenetics of human neuroblastoma cells in continuous culture. Cancer Res.

[31] Liang JC, Ning Y, Wang RY, Padilla-Nash HM, Schröck E, Soenksen D (1999). Spectral karyotypic study of the HL-60 cell line: detection of complex rearrangements involving chromosomes 5, 7, and 16 and delineation of critical region of deletion on 5q31. 1. Cancer Genet Cytogenet.

[32] Gimelbrant A, Hutchinson JN, Thompson BR, Chess A (2007). Widespread monoallelic expression on human autosomes. Science.

[33] Tang F, Barbacioru C, Nordman E, Bao S, Lee C, Wang X (2011). Deterministic and stochastic allele specific gene expression in single mouse blastomeres. PLoS ONE.

[34] Ni Y, Hall AW, Battenhouse A, Iyer VR (2012). Simultaneous SNP identification and assessment of allele-specific bias from ChIP-seq data. BMC Genetics.

[35] Giresi PG, Kim J, McDaniell RM, Iyer VR, Lieb JD (2007). Faire (formaldehyde-assisted isolation of regulatory elements) isolates active regulatory elements from human chromatin. Genome Res.

[36] Michailidou K, Hall P, Gonzalez-Neira A, Ghoussaini M, Dennis J, Milne RL (2013). Large-scale genotyping identifies 41 new loci associated with breast cancer risk. Nat Genet.

[37] Turnbull C, Ahmed S, Morrison J, Pernet D, Renwick A, Maranian M (2010). Genome-wide association study identifies five new breast cancer susceptibility loci. Nat Genet.

[38] Tuch BB, Laborde RR, Xu X, Gu J, Chung CB, Monighetti CK (2010). Tumor transcriptome sequencing reveals allelic expression imbalances associated with copy number alterations. PLoS One.

[39] Ross-Innes CS, Stark R, Teschendorff AE, Holmes KA, Ali HR, Dunning MJ (2012). Differential oestrogen receptor binding is associated with clinical outcome in breast cancer. Nature.

[40] Beerenwinkel N, Schwarz RF, Gerstung M, Markowetz F (2015). Cancer evolution: mathematical models and computational inference. Syst Biol.

[41] Morgan M, Pagès H, Obenchain V, Hayden N. Rsamtools: Binary alignment (BAM), variant call (BCF), or tabix file import. R package version 1.18.2. 2010. http://bioconductor.org/packages/release/bioc/html/Rsamtools.html.

[42] Lawrence M, Huber W, Pages H, Aboyoun P, Carlson M, Gentleman R (2013). Software for computing and annotating genomic ranges. PLoS Comput Biol.

[43] Langmead B, Trapnell C, Pop M, Salzberg SL (2009). Ultrafast and memory-efficient alignment of short DNA sequences to the human genome. Genome Biol.

[44] Sherry ST, Ward MH, Kholodov M, Baker J, Phan L, Smigielski EM (2001). dbSNP: the NCBI database of genetic variation. Nucleic Acids Res.

[45] Li H, Durbin R (2009). Fast and accurate short read alignment with Burrows–Wheeler transform. Bioinformatics.

[46] McKenna A, Hanna M, Banks E, Sivachenko A, Cibulskis K, Kernytsky A (2010). The genome analysis toolkit: a MapReduce framework for analyzing next-generation DNA sequencing data. Genome Res.

[47] DePristo MA, Banks E, Poplin R, Garimella KV, Maguire JR, Hartl C (2011). A framework for variation discovery and genotyping using next-generation DNA sequencing data. Nat Genet.

[48] Auwera GA, Carneiro MO, Hartl C, Poplin R, del Angel G, Levy-Moonshine A (2013). From FastQ data to high-confidence variant calls: the genome analysis toolkit best practices pipeline. Curr Protoc Bioinforma.

[49] Ward LD, Kellis M (2012). Haploreg: a resource for exploring chromatin states, conservation, and regulatory motif alterations within sets of genetically linked variants. Nucleic Acids Res.

