# Supplementary material for: BaalChIP: Bayesian analysis of allele-specific transcription factor binding in cancer genomes
Source: Genome Biol. 2017 Feb 24;18:39. doi: 10.1186/s13059-017-1165-7 (PMC5326502; doi:10.1186/s13059-017-1165-7)
Supplement: Additional file 1 — Supplementary Figures S1–S16. (PDF 6500 kb) [file 13059_2017_1165_MOESM1_ESM.pdf]

BaalChIP: Bayesian analysis of allelic imbalance in ChIP-seq data corrects for copy-number variation

de Santiago and Liu et al., 2017

University of Cambridge, Cancer Research UK, Robinson Way, CB2 0RE. Cambridge, UK. ^ Equal Contribution authors. * Corresponding authors

Additional File 1

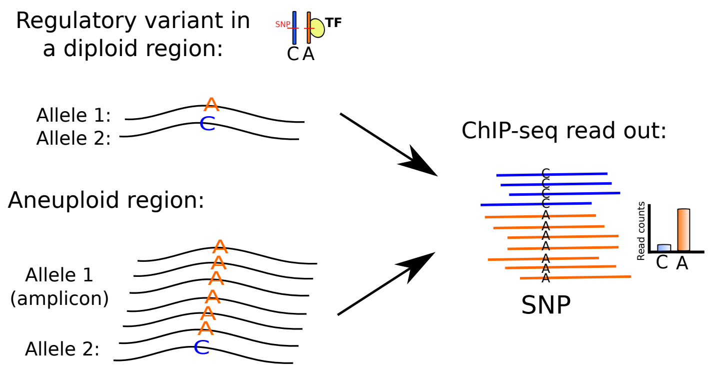

Figure S1: Example illustration of a ChIP-seq read out at a DNA-binding site when a true regulatory difference exists between two alleles (A allele depicted in orange and C allele depicted in blue), and when there is no regulatory difference between the two alleles. In a diploid sample, the protein (yellow circle) binds preferentially to the A allele. Here the regulatory effect is observed as an imbalance in the allelic ratios obtained from ChIP-seq read counts, with a higher number of reads carrying the A allele. In the presence of allele-specific copy number aberrations such as an amplicon affecting the A allele, the direct ChIP-seq readout may reflect the relative presence of the alleles, rather than the regulatory effect of the single nucleotide variant. Consequently, in the presence of copy number changes, ChIP-seq allelic ratios are not sufficient to uncover true cis-regulatory effects.

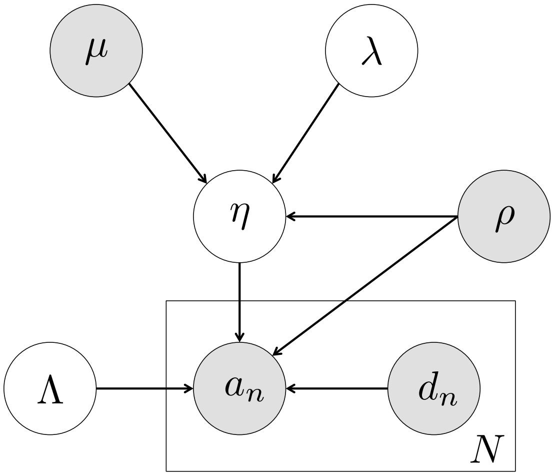

Figure S2: Graphical model of BaalChIP. The gray circles represent given variables and white circles are unknown variables. The direction of arrows indicates statistical dependency in the model. The observed reference allele read count is $\mathrm{a_n}$, total read counts is $\mathrm{d_n}$. n is the index of given factor (e.g. transcription factor) binding to the region of the SNP of interest. The rectangular plane and the capital N indicates that there are N factors binding to the SNP. The primary variable of interest is η representing allelic balance ratio. Λ is the precision parameter. The reference allele frequency at the SNP is ρ. $\mathrm{d_n}$, η, ρ, Λ are the parameters of a beta-binomial distribution that governs the uncertainty in $\mathrm{a_n}$. Finally, the reference mapping bias μ and λ are the mean and variance of a beta distribution which serves as a prior over η.

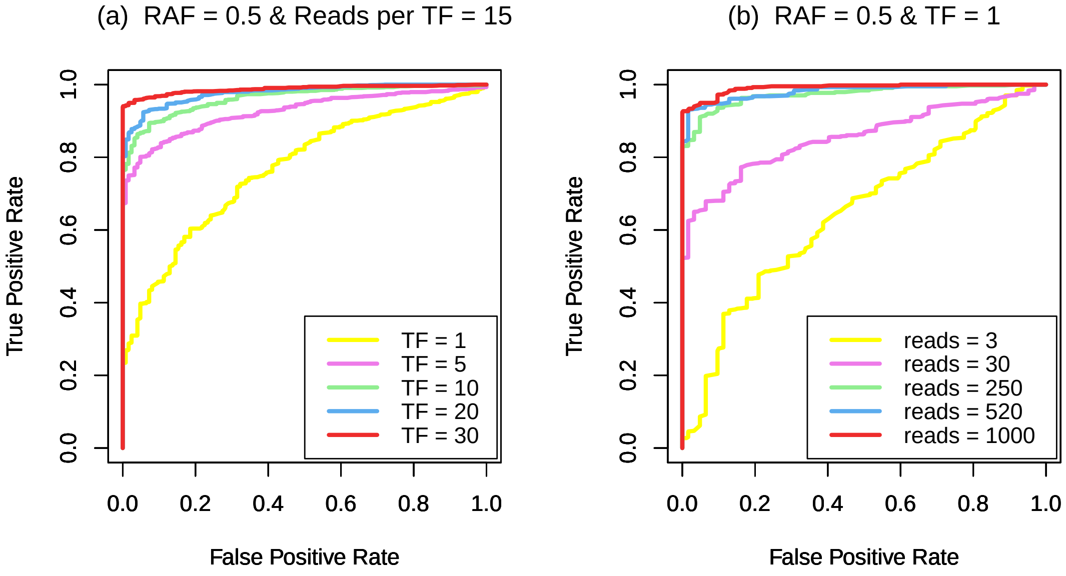

Figure S3: BaalChIP simulation performance with varying number of transcription factors (a) and read depth (b).

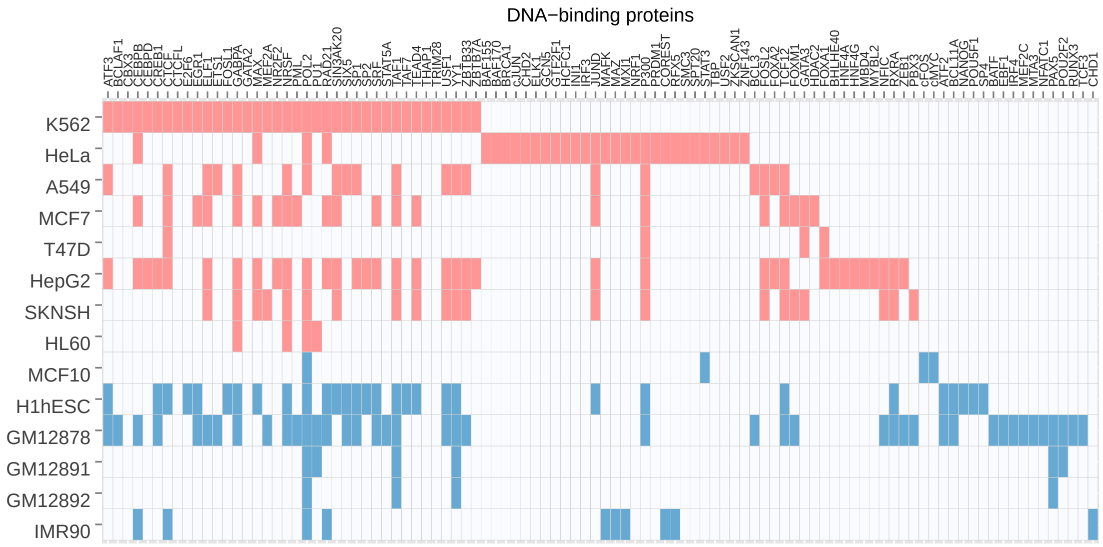

Figure S4: Data matrix summarizing the ENCODE samples used in this study for allele-specific binding analyses. Marks for cancer and non-cancer cell-lines are coloured in pink and blue, respectively. More detailed description including accession numbers can be found in Additional File 3: Table S2 and Additional File 4: Table S3.

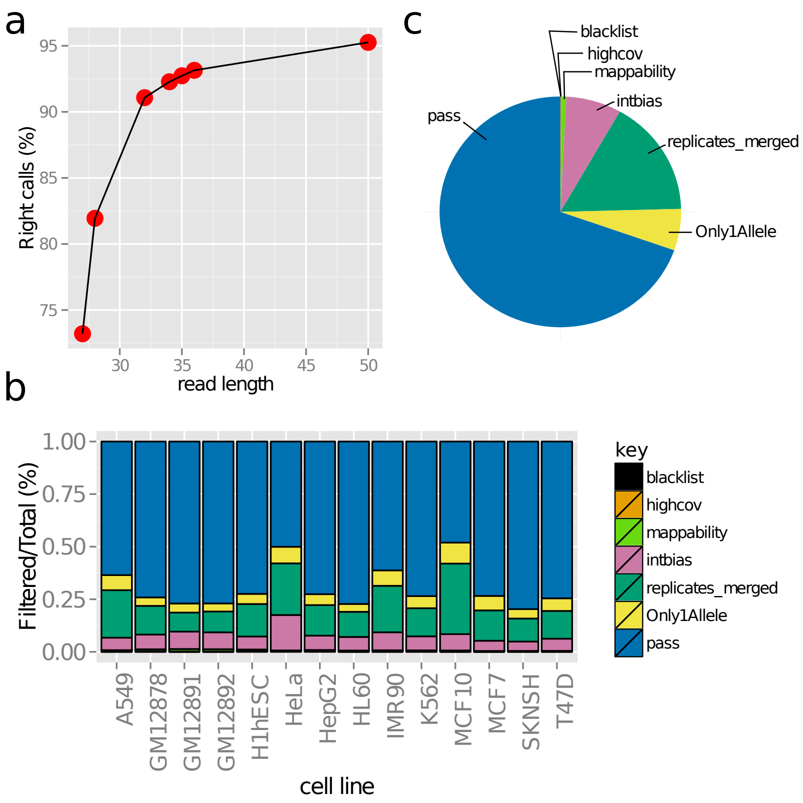

Figure S5: BaalChIP SNP filtering. (a) Percentage of SNPs with the correct number of aligned reads based on simulations of reads of different read lengths (28 to 50 mer). The percentage of correct calls increased with read length. (b) Proportion of SNPs that were filtered out in each filtering step. (b) The average proportion of SNPs that were filtered out in each filtering step. Average was calculated accross all 14 ENCODE cell lines.

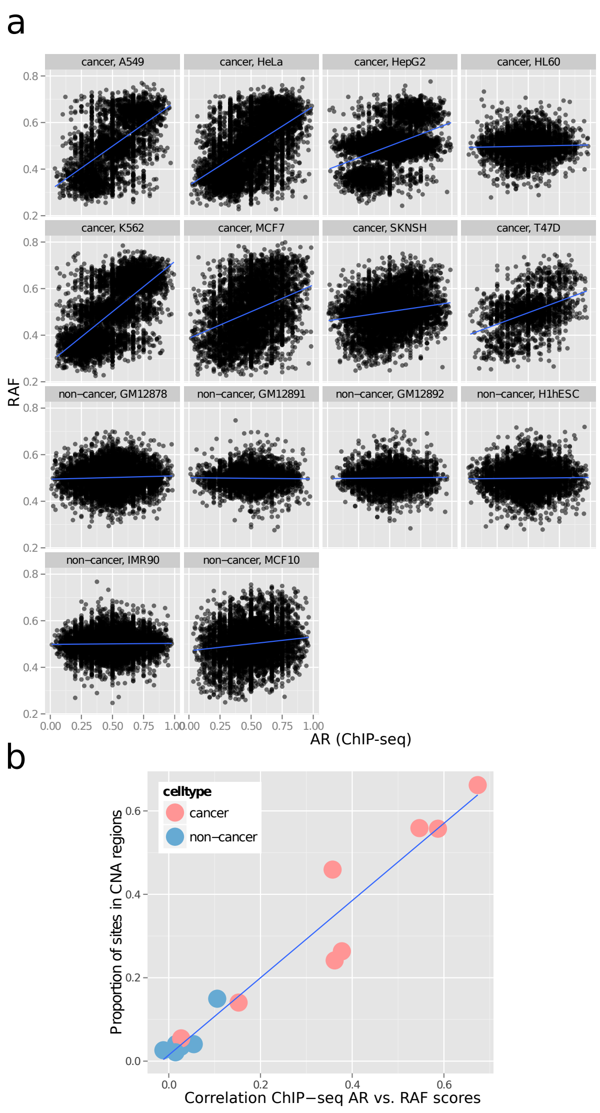

Figure S6: Correlation between the BAF values and the ChIP-seq allelic ratios (AR). (a) Scatter plots showing the correlation between RAF and AR at each SNP, for all considered cell lines of the ENCODE dataset. RAF corresponds to the BAF value with respect to the reference allele (RAF is equal to BAF if the reference allele corresponds to the B allele; RAF is equal to 1-BAF if the reference allele corresponds to the A allele). The blue line shows the fitted linear regression line. (b) Positive relationship between the Spearman correlation coefficient (obtained from the correlation of AR with RAF scores) and the proportion of sites in copy-number altered (CNA) regions. Each dot in the scatter plot corresponds to a cell line. Sites in copy number changed regions correspond to heterozygous SNPs with BAF score < 0.4 or BAF > 0.6.

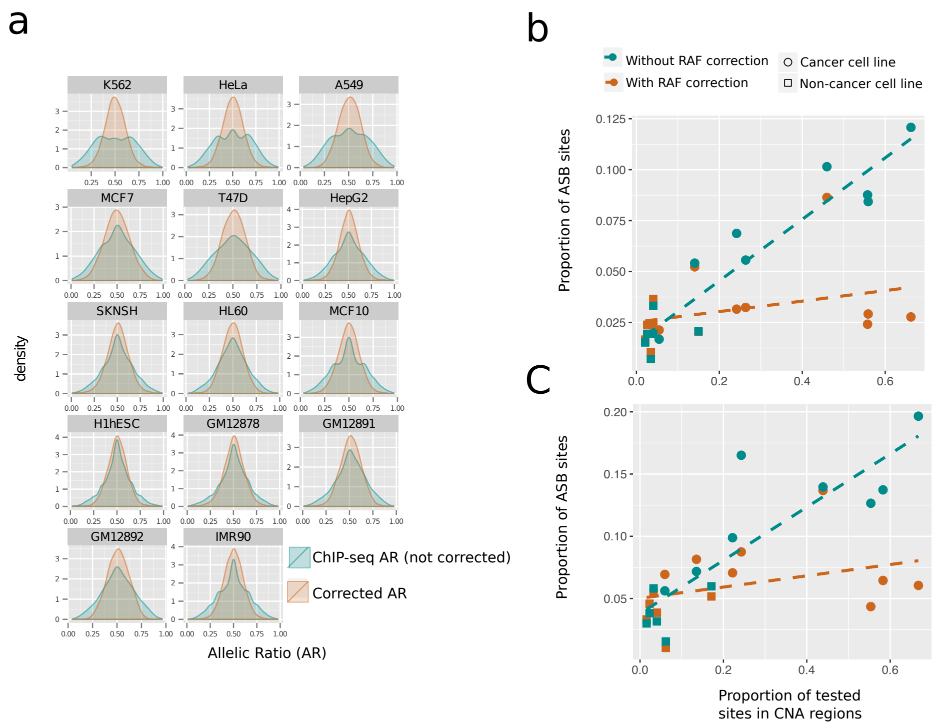

Figure S7: Effects of BaalChIP adjustment of the allelic ratios (AR) after correcting for the background Reference Allele Frequency (RAF) bias. (a) Density plots showing the distribution of allelic ratios before (green) and after (orange) BaalChIP correction. The ARs before correction were calculated directly from the ChIP-seq data and refer to the number of reads in the reference allele divided by the total number of reads. The corrected AR values were estimated by BaalChIP model after taking into account the RAF bias. The adjustment of AR is more dramatic in cancer cell lines. (b) Proportion of ASB sites detected with (orange) and without (green) RAF correction as a function of the proportion of sites within copy-number altered (CNA) regions. Each dot refers to a cell line. Sites in regions of copy number change refer to any SNP with a BAF score higher than 0.6 or lower than 0.4. Cancer cell lines have a higher proportion of SNPs in CNA regions and benefit more from BaalChIP correction. (c) Same as (b) but after selecting SNPs with 30X-40X coverage.

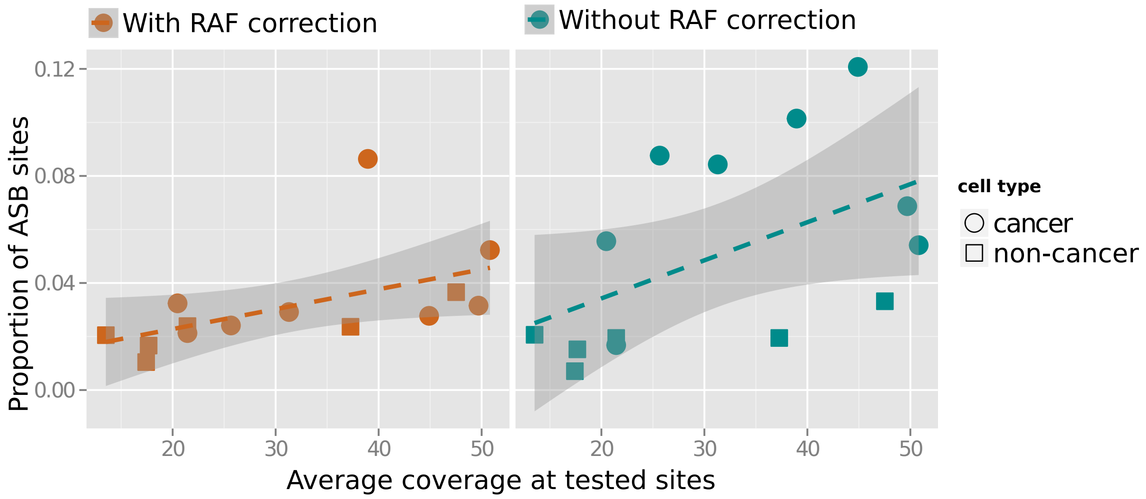

Figure S8: Proportion of identified ASB SNPs per cell line. Correlation between the proportion of identified ASB SNPs and the average coverage at all assayed heterozygous SNPs. The general correlation is expected due to the higher power to detect ASB when coverage is high.

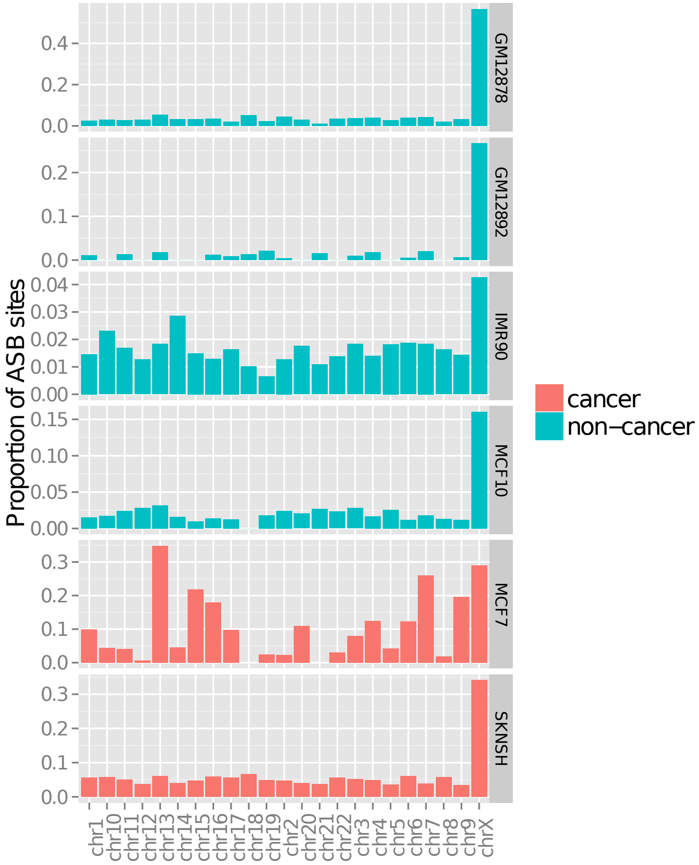

Figure S9: Higher rates of ASB on chromosome X of female cell lines than in autosomal chromosomes.

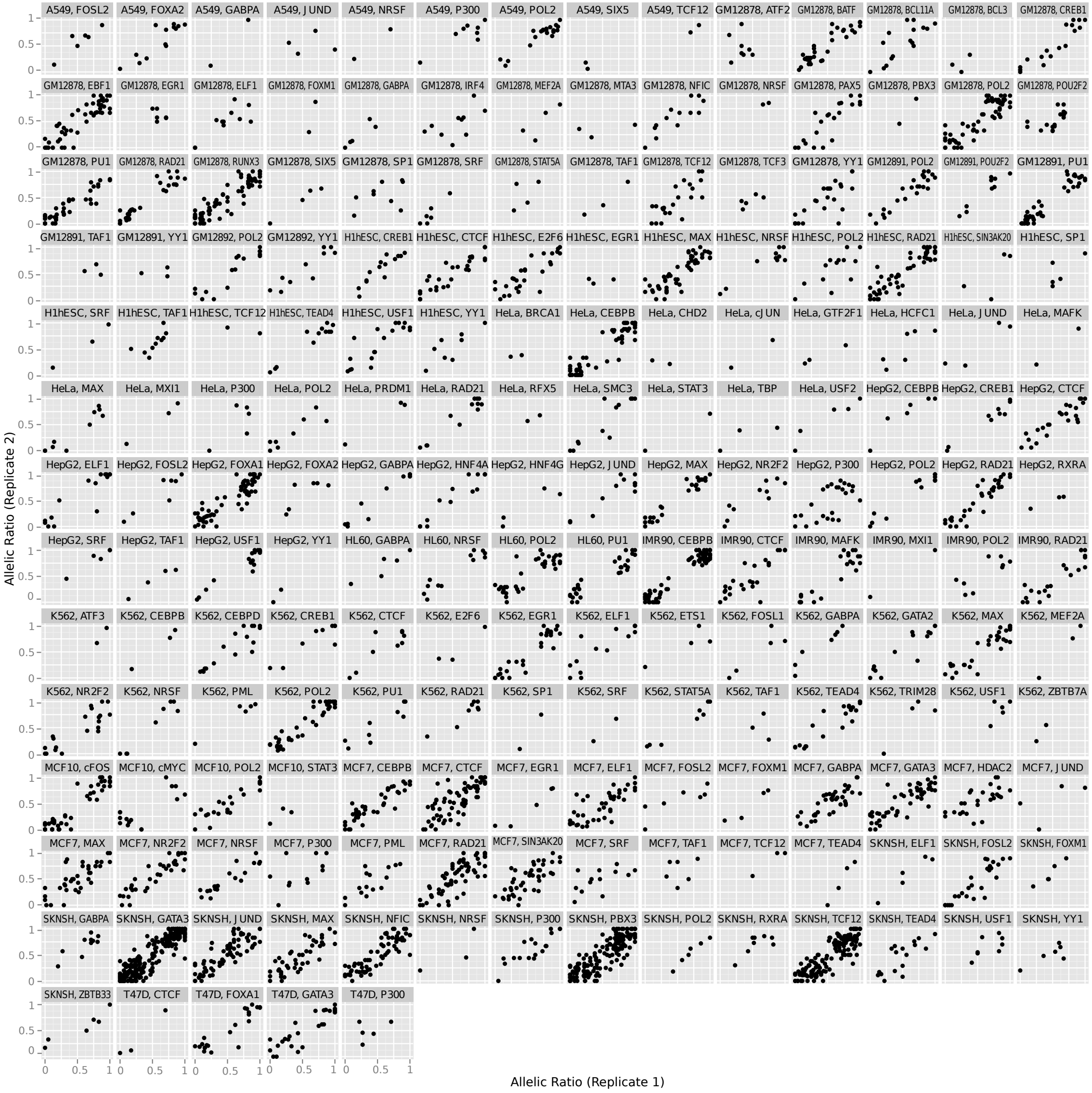

Figure S10: Consistency of ASB across biological replicates. Each dot in the scatter plot represents the allelic ratio at a single SNP obtained for two replicated samples from the same ChIP-seq experiment.

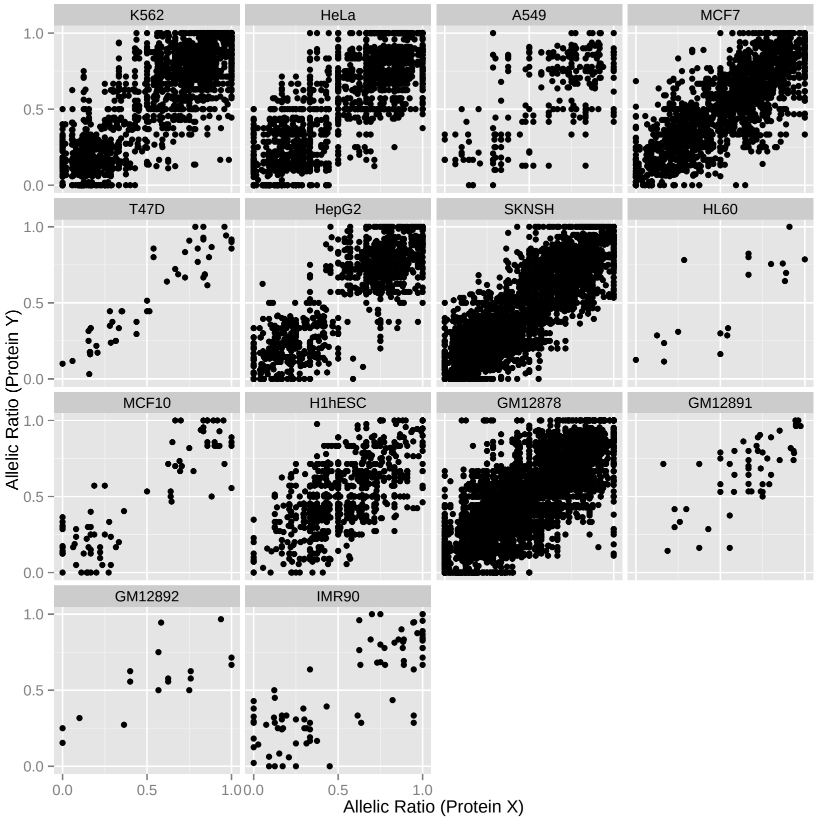

Figure S11: Consistency of ASB across pairs of different assayed proteins (Protein "X" and "Y"), within the same cell line. Each dot in the scatter plot represents the allelic ratio at a single SNP obtained for distinct ChIP-seq experiments in the same cell line (i.e distinct ChIPed proteins bound at the same site).

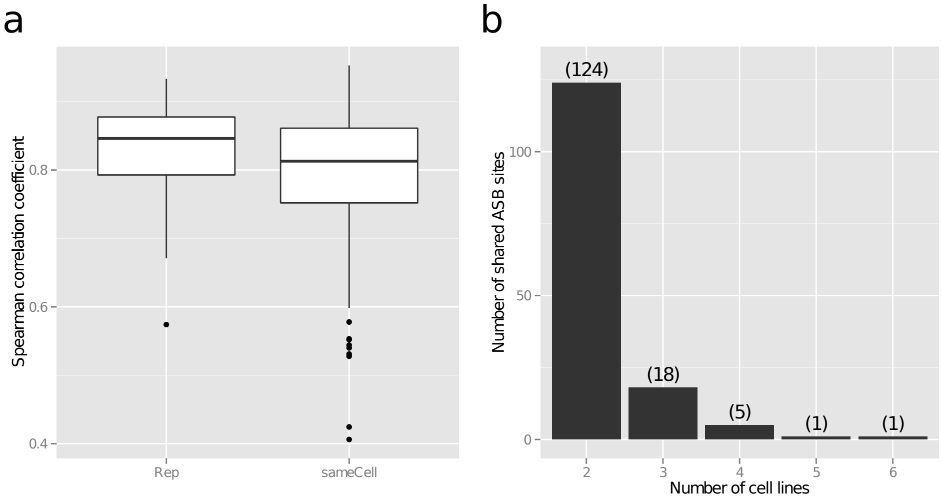

Figure S12: (a) Distribution of Spearman correlation coefficients obtained for the correlation of allelic ratios obtained for pairs of biological replicates (Rep), and between distinct proteins bound at the same site (sameCell). The allelic ratios are highly correlated in both scenarios. (b) Number of shared ASB SNPs across cell lines. Numbers in parenthesis show the number of ASB SNPs shared between 2 to 6 cell lines. 149 ASB SNPs (124+18+5+1+1) were concomitantly detected in two or more cell lines.

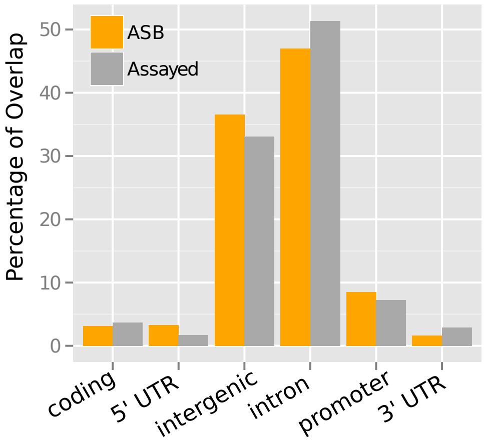

Figure S13: Percentage of ASB SNPs overlapping gene annotations (5' UTR, 3' UTR, coding, intergenic, intron or promoter). The largest proportion of ASB SNPs was found in introns and intergenic regions. A similar distribution pattern is observed when considering all assayed heterozygous SNPs.

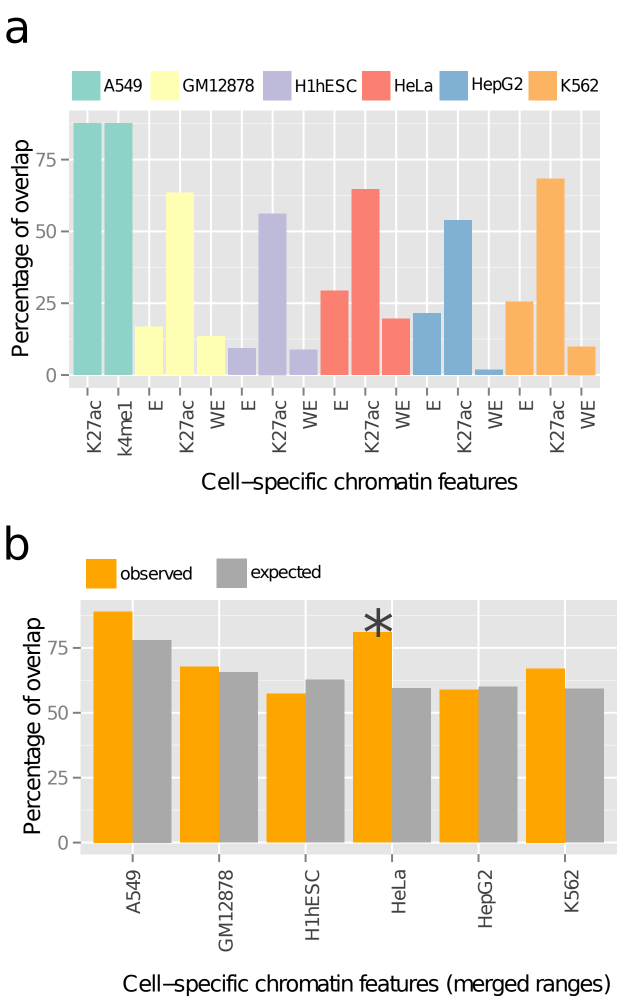

Figure S14: Percentage of ASB SNPs overlapping putative enhancer regions from the matched cell line. (a) Putative enhancer regions were retrieved from publicly available ENCODE datasets on H3K27ac and H3K4me1 modified regions, and previously predicted weak enhancers (WE) and strong enhancer (E) sites obtained from Segway chromatin state annotations of the ENCODE data. (b) Comparison of the observed and expected number of ASB SNPs that map to enhancer regions. A significant difference (*) was only detected in HeLa cells.

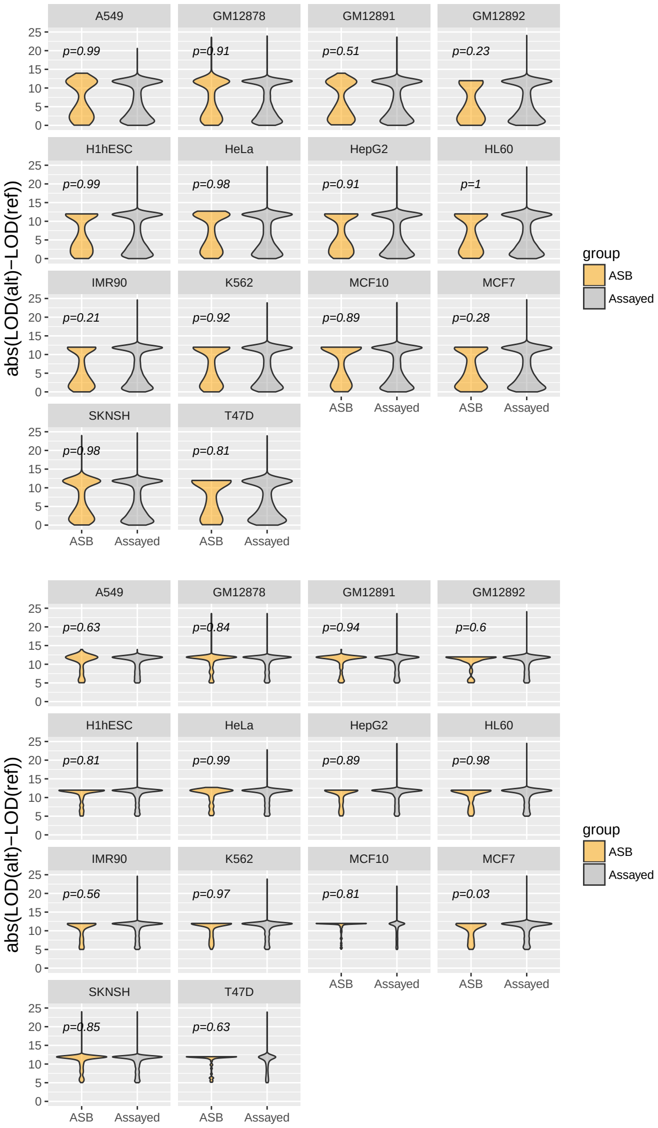

Figure S15: Distribution of absolute DELTA scores (deltaLOD) of motif-disrupting SNPs with (a) deltaLOD > 0 and (b) deltaLOD > 5. The DELTA score corresponds to the change in the PWM score between SNP alleles (DELTA = LOD(ref) - LOD(alt)). P-values correspond to the Kolmogorov-Smirnov test when comparing the distribution of DELTA scores between motif-disrupting ASB SNPs with all motif-disrupting SNPs.

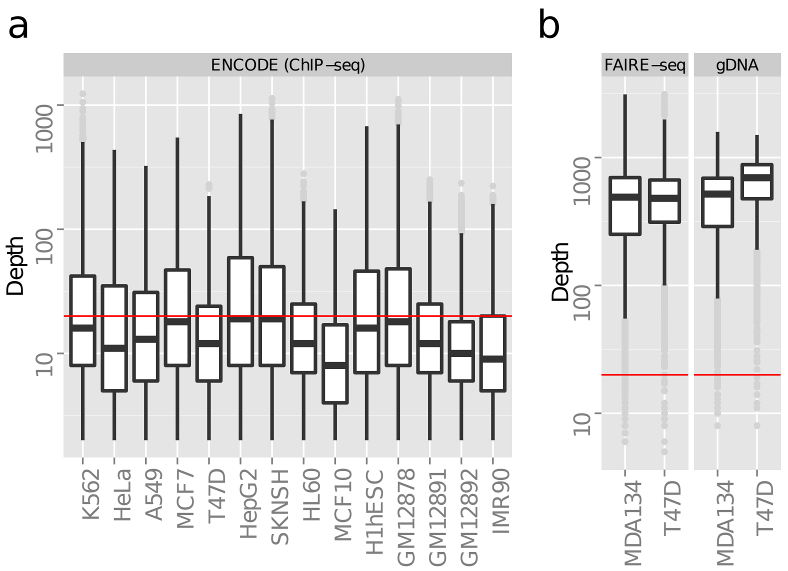

Figure S16: Distribution of depth of coverage at tested heterozygous SNPs in (a) ENCODE ChIP-seq datasets (pooled data across all ChIP-seq samples in each cell line) and (b) targeted sequencing FAIRE-seq datasets (pooled data for three replicates).
